# Swedish Chronic Pain Biobank: protocol for a multicentre registry and biomarker project

**DOI:** 10.1136/bmjopen-2022-066834

**Published:** 2022-11-30

**Authors:** Bijar Ghafouri, Malin Ernberg, Paulin Andréll, Emmanuel Bäckryd, Marcelo Rivano Fisher, Yvonne Freund-Levi, Henrik Grelz, Olaf Gräbel, Rolf Karlsten, Eva Kosek, Monika Löfgren, Åsa Ringqvist, Karin Rudling, Britt-Marie Stålnacke, Niklas Sörlén, Karin Uhlin, Hans Westergren, Björn Gerdle

**Affiliations:** 1 Pain and Rehabilitation Centre, and Department of Health, Medicine and Caring Sciences, Linköping University, Linkoping, Sweden; 2 Department of Dental Medicine, Karolinska Institutet, and the Scandinavian Center for Orofacial Neurosciences (SCON), Karolinska Institute, Stockholm, Sweden; 3 Region Västra Götaland, Sahlgrenska University Hospital, Östra, department of Anaesthesiology and Intensive Care Medicine, Pain Centre, Sahlgrenska Academy, Gothenburg, Sweden; 4 Department of Anaesthesiology and Intensive Care Medicine, Institute of Clinical Sciences at Sahlgrenska Academy, University of Gothenburg, Gothenburg, Sweden; 5 Department of Neurosurgery and Pain Rehabilitation at Skåne University Hospital and Faculty of Medicine Department of Clinical Sciences Malmö, Lund University, Lund, Sweden; 6 Rehabilitation Medicine Research Group, Department of Health Sciences, Lund University, Lund, Sweden; 7 School of Medical Sciences, Örebro University and department of Geriatrics, University Hospital Örebro, Örebro, Sweden; 8 Department of geriatrics, Södertälje Hospital, Södertälje, Sweden; 9 Department of Clinical Science and Education, Södersjukhuset, Karolinska Institutet, Stockholm, Sweden; 10 Department of Surgical Sciences, Anaesthesiology and Intensive Care, Uppsala University Hospital, Uppsala, Sweden; 11 Department Surgical Sciences, Uppsala University, Uppsala, Sweden; 12 Department of Clinical Neuroscience, Karolinska Institutet, Stockholm, Sweden; 13 Department of Clinical Sciences, Karolinska Institutet, and Department of Rehabilitation Medicine, Danderyd Hospital, Stockholm, Sweden; 14 Department of rehabilitation medicine, University hospital Örebro, Örebro, Sweden; 15 Department of Community Medicine and Rehabilitation, Rehabilitation Medicine, Umeå University, Umeå, Sweden; 16 Department of Clinical Science, Neurosciences, Umeå University, Umeå, Sweden

**Keywords:** rehabilitation medicine, pain management, neurological pain, back pain

## Abstract

**Introduction:**

About 20% of the adult population have chronic pain, often associated with psychological distress, sick leave and poor health. There are large variations in the clinical picture. A biopsychosocial approach is used in investigation and treatment. The concept of personalised medicine, that is, optimising medication types and dosages for individual patients based on biomarkers and other patient-related factors, has received increasing attention in different diseases but used less in chronic pain. This cooperative project from all Swedish University Hospitals will investigate whether there are changes in inflammation and metabolism patterns in saliva and blood in chronic pain patients and whether the changes correlate with clinical characteristics and rehabilitation outcomes.

**Methods and analysis:**

Patients at multidisciplinary pain centres at University Hospitals in Sweden who have chosen to participate in the Swedish Quality Registry for Pain Rehabilitation and healthy sex-matched and age-matched individuals will be included in the study. Saliva and blood samples will be collected in addition to questionnaire data obtained from the register. From the samples, proteins, lipids, metabolites and micro-RNA will be analysed in relation to, for example, diagnosis, pain characteristics, psychological distress, body weight, pharmacological treatment and clinical rehabilitation results using advanced multivariate data analysis and bioinformatics.

**Ethics and dissemination:**

The study is approved by the Swedish Ethical Review Authority (Dnr 2021–04929) and will be conducted in accordance with the declaration of Helsinki.

The results will be published in open access scientific journals and in popular scientific relevant journals such as those from patient organisations. Data will be also presented in scientific meetings, meeting with healthcare organisations and disseminated in different lecturers at the clinics and universities.

**Strengths and limitations of this study**

- The major strength of this study is that both omics methods and patient-reported outcomes measure from the Swedish Quality Registry for Pain Rehabilitation will be used.
- Researchers from different professions with strong research backgrounds in chronic pain, rehabilitation and omics collaborate in this pain precision medicine study.
- There is a risk of heterogeneity of the samples as this study is a multicentre study in a large geographical area where the infrastructure for data collection is different.
- Individuals not able to read and write Swedish are excluded, which make the results less generalisable.

## Introduction

About 20% of the adult population lives with at least moderate–severe chronic pain; often with concomitant complex psychological distress including depression and anxiety. Pain will also reduce patients’ ability to study or work full time, they will show higher levels of sick leave and show reduced quality of life.^1^ There are large variations in the clinical picture in chronic pain patients because of the presence of different comorbidities. For example, psychiatric comorbidities are common in chronic pain and about 40% of patients managed at specialist level have comorbid symptoms of depression and/or anxiety.^2^ More than 20% of years lived with disabilities—a measure of non-fatal disabilities—are caused by pain conditions both in Sweden and globally.^3^

*Acute* tissue damage initiates plastic and reinforcing mechanisms in the pain system peripherally and centrally, which in interaction with psychological and social factors create the experience of pain at a given moment.^4^ Chronic pain in combination with depression constitutes a large part of the pain complex, why depression also must be considered when diagnosing the patient. Moreover, positive correlations exist between depressive symptoms and pain intensity. Social factors such as working conditions can be risk factors for the chronification of pain, and when chronic pain has developed return to work will be more difficult (ie, chronic pain has social consequences). Modern pain care is, therefore, based on a bio–psycho–social approach to diagnosis and treatment.^5 6^

*Chronic pain* is not an acute pain that persists over time, but further plastic neurobiological changes occur in interaction with psychological and social factors. Imaging techniques have provided an in-depth understanding of how the brain processes and creates the experience of pain. Chronic pain is associated with chemical changes in (1) structures in the brain that process pain, (2) the physiological interactions between these structures, (3) the descending control of nociception and (4) central hyperexcitability (central sensitisation).^7 8^

Chronic widespread pain (CWP) including fibromyalgia (FM) is the extreme of complex pain conditions. FM is characterised by altered nociception and is a prototype of a nociplasic pain condition; however, it is well known that patients initially suffering from nociceptive pain can in time develop nociplastic pain conditions.^9^ It has been argued that CWP/FM is a typical example of a central pain condition, that is, that peripheral factors have little or no role. Others believe that peripheral factors initiate and perpetuate the central changes in analogy with, for example, coxarthrosis where central changes are normalised after hip replacement.^10^

In various studies, we have shown an increased presence of pain-mediating (eg, glutamate, serotonin), metabolic (eg, lactate and pyruvate) and analgesic (eg, N-acylethanolamines (NAE)) substances in muscle in neck–shoulder pain and in CWP/FM.^11^ Other researchers have recently shown changes in the peripheral nociceptors of FM^12^ and blood biomarkers in chronic pain.^13 14^ Proteomic studies are increasingly being performed in the field of pain medicine.Using targeted and untargeted proteomics, our research group has found significant differences in the protein/inflammation pattern in muscles and plasma in CWP/FM.^15–17^ Such changes also correlate with, for example, pain intensity, pain sensitivity and psychological strain.^15 18^ Taken together, this provides support for peripheral factors that significantly contribute to the maintenance of CWP/FM.

In the literature, it is suggested that low-grade peripheral or systemic inflammation perpetuates chronic pain and is also involved in psychological/psychiatric conditions such as depression and obesity.^19 20^ Various proinflammatory cytokines and chemokines have been studied. However, the literature is not consistent; in a large FM study, we found no changes in ‘classic’ proinflammatory cytokines in muscle or plasma.^21^ Two recent systematic reviews of peripheral cytokines and chemokines—mainly based on single or few molecules—have not been consistent.^22 23^ Such hypothesis-driven studies have generally focused on a few molecules, while there are only a few exploratory studies of a larger number of molecules (panels). Low-grade inflammation/neuroinflammation has also been studied in neuropathic pain conditions.^24–26^

Preliminary biomarker data available in the literature need to be confirmed in larger studies that reflect the normal flow of chronic pain patients where/in which comparisons are made between pain diagnoses and that include control for sex, pain characteristics, comorbidity, body weight and pharmacological therapy. The inflammation pattern according to ‘classic’ inflammation markers seems to be of preliminary importance for the treatment outcome in depression^27^ and obesity, as well as for psychological treatment, such as cognitive behavioural therapy (CBT) in chronic pain.^28^ Interdisciplinary Pain Rehabilitation Programs (IPRPs), in which psychological treatment (including CBT) and physical activity and exercise are important components, constitute evidence-based treatment for chronic pain but with small to moderate effect sizes.^29–32^ It has not yet been considered if activated biological mechanisms may impact on the effect of IPRP. It is, therefore, important to investigate whether IPRP result is related to inflammation and metabolism patterns. To the best of our knowledge, there are no reports on established pain biobank that combine health informatics with biomarkers and bioinformatics to study chronic pain mechanisms in everyday life patients.

## Aim and hypotheses

The aim of this multicentre project is to investigate whether there are differences in inflammation and metabolism patterns between common chronic pain diagnoses and to sex-matched and age-matched healthy controls from the general Swedish population. Further aims are to characterise and compare the changes of the grade of inflammation, and metabolism in different diagnostic groups of chronic pain patients regarding, for example, sex, diagnosis, pain, pain sensitivity, comorbidity pattern, body weight, pharmacological therapy and clinical rehabilitation outcomes.

The hypotheses are:

1. There are unique biomarker signatures in plasma and saliva that distinguish common chronic pain diagnoses from each other and from healthy controls.
2. There are significant correlations between the identified biomarker profiles and pain characteristics, for example, pain intensity, pain sensitivity and anatomical spread of pain, comorbidity patterns, body weight and pharmacological therapy, and clinical rehabilitation outcomes.

## Methods

### Participants

**Figure 1:**
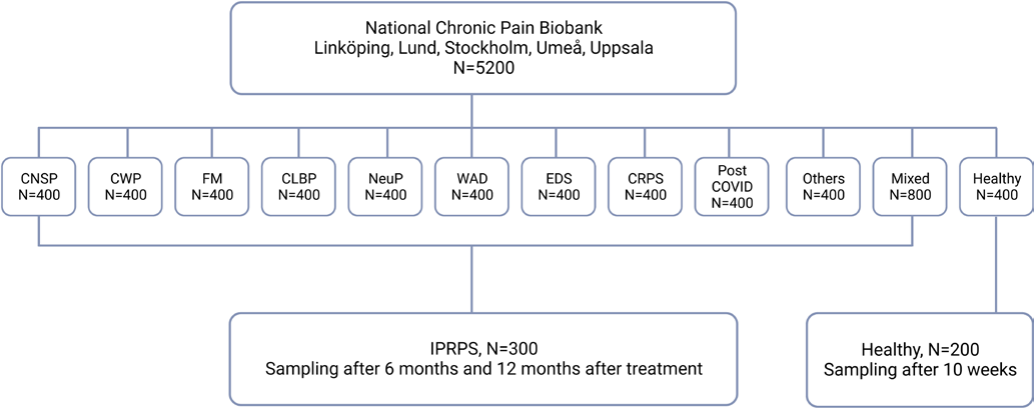
Flowchart of the study design outlining numbers of subjects in each chronic pain group and healthy controls. CNSP, chronic neck and shoulder pain; CWP, chronic widespread pain; FM, fibromyalgia; CLBP, chronic low back pain; CRPS, Complex regional pain syndrome; EDS, Ehlers-Danlos syndrome; IPRP, Interdisciplinary Pain Rehabilitation Program; WAD, whiplash disorder.

This multicentre project will include patients (>18 years) with chronic pain who are recruited from the *Swedish Quality Registry for Pain Rehabilitation* (SQRP) and healthy sex-matched and age-matched controls from university hospital at Linköping, Stockholm, Uppsala, Umeå, Gothenburg and Lund; see figure 1.

### Patients with chronic pain

The project will mainly use patient-reported data from SQRP (www.ucr.uu.se/nrs/) for the participating university clinics. The project leader (Björn Gerdle) is the research leader for the national research group for SQRP and a member of its steering group.

SQRP is based on *Patient-reported outcome measures* in the form of a written questionnaire. Validated instruments are used in the survey and collectively capture the various facets of the International Classification of Functional Conditions, Disabilities and Health. The survey includes background variables (*age, sex, country of origin, education and healthcare consumption, weight, height, pain duration and pain extent (according to a list of 36 anatomical regions*)) and pain characteristics, degree of FM-ness, psychological distress, difficulty sleeping, pain coping, fear of movement, consequences of living with chronic pain, physical activity level as well as health-related quality of life and perceived health (table 1, online supplemental appendix 1). The response rate is >90% in SQRP, which can be interpreted as patients considering it important to be able to share how they experience their situation. The current diagnoses according to ICD-10 are also registered in SQRP.

10.1136/bmjopen-2022-066834.supp1 Supplementary data

**Table 1:** Patient-reported outcome measures to be used in the project

Survey	Instrument/scales	Properties
Pain characteristics		
Intensity	NRS (0–10) MPI Pain intensity (0–6)	0=no pain, 10=worst pain experienced Based on two items, each scored 0–6
Interference	MPI-Pain Interference (0–6)	Based on 11 items, each scored 0–6
Fibromyalgia	2016 criteria	Widespread pain index (WPI): 0–19 sites Symptom severity score (SSS): 0–12 FMness score: WPI+SSS.
Psychological distress		
Depression	HADS-Depression (0–21)	Sum of 7 items, each scored 0–3
Anxiety	HADS-Anxiety (0–21)	Sum of 7 items, each scored 0–3
	MPI-Affective distress (0–6)	Based on 3 items each scored 0–6
Pain catastrophising	PCS (0–52)	Sum of 13 items, each scored 0–4; three subscales can be obtained
Difficulty sleeping		
	ISI (0–28)	Sum of 7 items, each scored 0–4
Pain coping		
	CPAQ8 (0–48)	Sum of 8 items, each scored 0–3; two subscales can be obtained
Fear of movement		
	TSK (0–68)	17 items, each scored 0–4 according to agreement with statement and summed
Impact of chronic pain		
Control of life	MPI-Life control (0–6)	Based on 4 items, each scored 0–6
Social support	MPI-Social support (0–6)	Based on 2 items, each scored 0–6
Health-related quality of life		
	RAND-36 (0–100)	36 items in eight subcategories scored varying between 0 and 100% (with fixed intervals). The mean for each subscale is calculated
	EQ-5D	Consists of two parts. The first part is an index obtained from five dimension items scored 0–5. The second part is self-estimation of today’s health according to a 100-point thermometer-like scale (EQ5D-VAS) with defined end points (high values indicate better health and low values indicate worse health)
Physical activity		
Sedentary behaviour		Minutes per week
Low intensity		
Moderate intensity		
Vigorous intensity		

CPAQ8, Chronic Pain Acceptance Questionnaire based on eight items; HADS, Hospital Anxiety and Depression Scale; ISI, Insomnia Severity Index; MPI, Multidimensional Pain inventory Swedish version; NRS, numeric rating scale; PCS, Pain Catastrophizing Scale; RAND-36, modernised version of Short-Form Health Survey-36; TSK, Tampa scale for Kinesiophobia.

The patients who refer to the clinics respond to the SQRP questionnaire according to table 1 in connection with clinical assessment. Patients participating in IPRP also answer questionnaires immediately after and 12 months after rehabilitation.

All patients who respond to SQRP at the relevant university clinics will be asked to participate in the study and may submit saliva samples and blood samples (see below). In this project, we will study the following 11 conditions/diagnoses (based on the manual produced within SQRP by, among others, the project leader):

1. Chronic neck–shoulder pain (cervicalgia, trapezius myalgia).
2. Chronic low back pain without sciatica.
3. Generalised pain (CWP) according to criteria ACR 2016 criteria.
4. FM according to the ACR (american college of Rheumatology) 2016 criteria.
5. Neuropathic pain including sciatica.
6. Chronic whiplash disorders.
7. Ehlers-Danlos syndrome or hypermobility syndrome.
8. Complex regional pain syndrome.
9. Pain post-COVID infection.
10. Chronic pain, not elsewhere classified (ICD R52.1, R52.2 and R52.9).

In addition, we will investigate the patterns when several pain diagnoses are present simultaneously.

### Healthy controls

Age-matched and sex-matched healthy control persons will be recruited via advertisements and answer a customised version of the SQRP questionnaire. Saliva and blood samples will be collected. Half of the healthy controls will be asked to submit new samples after about 10 weeks.

### Exclusion criteria for patients and controls

Coagulation disorders with predisposition to bleeding, medication with anticoagulants (low-dose aspirin is permitted), hypersensitivity to anaesthetic, serious psychiatric disease (investigator’s judgement, eg, psychosis or suicidal ideation) and difficulties in understanding the Swedish language will be excluded.

### Supplementary survey

In addition to the instruments included in the SQRP (or equivalent adapted for healthy controls), the participants will also answer a brief questionnaire in close connection with the biofluid sampling. This charts the time of sampling, fasting or not, current medication and health food preparations (eg, curcumin), pain intensity and present and previous illnesses, for example, diabetes.

### Biofluid sampling

Sampling consists of saliva and blood samples drawn in the morning and during fasting. Saliva samples will be taken with Salivette (Sarstedt AG & Co, PO Box 1220, D-51582, Nümbrecht, Germany, obtained from VWR, article number: 1 01 093–968). The participants will be asked to avoid brushing teeth or drinking (water is ok) at least 1 hour before sampling. They rinse their mouth with water and wait for 15 min before taking the swab in the mouth. After 3 min, they spit the swab into the salivette tube containing a protease cocktail inhibitor. The salivette will be centrifuged (5 min, 1000× g) and the supernatant is transferred to a new tube and aliquots into 200 µl in 0.6 mL eppendorf tubes and will be stored at −86°C until analysis. The swab will be stored at −86°C for future cell extraction. The total volume of saliva will be recorded together with time of sampling, any complication, for example, bleeding, shorter time than 3 min due to uncomfortable feelings to have the swab in the mouth.

Blood samples (2×8.5 mL) will be collected in P100 tubes (article number: 366448, BD Diagnostics System, Frankling Lakes, New Jersey) according to the manufacture’s recommendations. The sample will be centrifuged at 2500 g for 20 min at room temperature within 2–4 hours. Plasma will be extracted by carefully removing the upper part of the supernatant in fractions to a 10 mL tube, and after mixing gently will be aliquoted into 200 µl in 0.6 mL eppendorf tubes and stored at −86°C. The cell fraction will be removed to a new tube and stored at −86°C. The time for blood sampling and centrifugation, any signs of hemolysis, any complication with sampling will be recorded.

The samples will be handled according to previously developed methodology.^16 17^

### Biochemical analyses

The analyses that will be used in this study are mainly exploratory omics analysis, that is, which proteins, metabolites and lipoproteins will be identified cannot be determined in advance. The following analyses will be performed according to the methodology previously described by our research group.^15 17 26^

- Exploratory analyses using panels for inflammation, cytokines and chemokines and neuroinflammation comprising many proteins (from Olink Bioscience, Uppsala^15^ and from Meso Scale Discovery).^26^
- Exploratory proteomics analysis using 2D gel electrophoresis and/or shotgun proteomics.^16 33^
- Exploratory metabolomic and lipoprotein profile including free fatty acids.
- Antinociceptive substances, that is, endocannabinoids, NAE, endorphins, alpha-amylase, and cortisol.
- Metabolites such as lactate, pyruvate, glutamate and other single pain-mediating molecules such as substance P, bradykinin, serotonin and BDNF (brain-derived neurotrophic factor).
- Pending analysis, the samples are stored in the biobank facilities with which the participating university clinics are associated.

### Data from SQRP

The surveys in SQRP, including the supplementary questionnaire, will include a careful characterisation of the patients and the healthy controls. In the correlation analyses between biomarkers and pain characteristics, the following variables will initially be used:

- Diagnosis (from manual produced within SQRP).
- Pain intensity.
- Extent of pain spreading/pain localisation.
- Pain duration.
- Psychologic distress (depression, anxiety, pain catastrophising, fear of movement and insomnia).
- Sex.
- Body mass index (BMI).
- Physical activity level.
- Pharmacological therapy and health food preparations.

### Interdisciplinary multimodal rehabilitation programme

The inclusion criteria will be decided by the clinical examination and screening questionnaires concerning both anthropometric measures as well as subjective health followed by an inter professional conference between the medical doctor, a psychologist, a physiotherapist and an occupational therapist. The IPRP team makes the decision which patients they believe would benefit from the IPRP, in accordance with recommendations from the Swedish Agency for Health Technology Assessments and Assessment of Social Services. The patient should be motivated and have the potential for an active change to be included in IPRP. All patients will participate in a specific individual exercise with a physiotherapist (with both relaxation therapy and strengthening specific regions of the body to unburden), individual therapy and group therapy led by a clinical psychologist (CBT, education, coping and mindfulness) as well as environmental work changes from an occupational therapist, working with education and return to work strategies in group of six to nine persons, commonly 20 hours per week of group-based activities for 6–8 weeks.^34 35^ Pain education (including lectures in basic pain science and pain management) will be offered both for patients as well as for their relatives, friends and colleagues. For descriptions and content of the Swedish IPRPs, see Gerdle *et al*, Ringqvist *et al* and Gerdle *et al.*^36–38^

### Statistics

In addition to descriptive statistics, advanced multivariate statistics (MVDA^18^; that is, advanced principal component analysis and orthogonal partial least square regressions) will be used according to the guidelines presented by Wheelock and Wheelock for omics data^39^ using SIMCA-P + (Sartorius Stedim Biotech, Umeå, Sweden). The methods are necessary to manage and take advantage of the intercorrelation pattern (ie, multicollinearity) between the identified substances. MVDA enables analyses where the number of variables is significantly greater than the number of observations (ie, short and broad data tables), which more traditional multivariate regressions cannot easily handle. The research group has a long experience of the methods and has used these in previous studies and has an established collaboration with statisticians.

### Sample size calculation

There are limited numbers of study that report methods for determining the sample size at MVDA.^40^ In the literature, for example, proteomic and metabolomic studies for chronic pain have so far been rather small and usually include 20+20 participants. The literature points out the need for large cohorts, especially in complex disease states such as chronic pain, and that the results are replicated in new cohorts. Here, we make the assessment that at least 200 patients (from both sexes) in each of the 10 diagnostic groups (see above) must be recruited and the same number for replication of the results. In the group with several diagnoses, the heterogeneity is significant, so double the number is deemed necessary. The numbers are required to get a sufficient spread in terms of, for example, psychological load, BMI and pharmacotherapy and to be able to identify clinically relevant subgroups within a diagnosis. Four hundred healthy controls (even sex distribution) are deemed necessary; half of these leave new samples after about 10 weeks. For analyses of the relationships with treatment results (mixed diagnoses), 300 patients are considered necessary.

In summary, we intend to recruit a total of 400 patients in each diagnostic group (1–10) and 800 in mixed diagnostic group for analysis at one time, 300 patients participating in IPRP treatment will leave samples immediately before the treatment period and are followed up on two occasions and 400 healthy controls. This means that 4800 patients and 400 healthy controls, that is, a total of 5200 participants, will be involved.

### Patient and public involvement

Neither patients nor the public are involved in the design, conduct, reporting or dissemination plans associated with this research.

## Discussion

Sweden has a population of about 10 million people, and the societal costs of approximately 20% of the adult population having moderate–severe chronic pain have been calculated by the Swedish Agency for Health Technology Assessments and Assessment of Social Services in 2003 as SEK 87 billion per year, corresponding to approximatively 1 billion US dollars per million inhabitants and year. Chronic pain is often associated with extensive suffering and poor health. At the same time, it is necessary to state that the effects of, for example, pharmacological treatment are limited; a maximum of 25%–30% of the patients report significant and clinically valuable effects. The effect sizes for IPRP are only small to moderate according both to systematic reviews and to registry studies of real-world patients.^37 41^

The importance of developing mechanism-based diagnostics and treatment for chronic pain has been emphasised.^42^ Pain clinicians largely lack precision medicine tools that can provide support for treatment choice. This constitutes a significant lack of knowledge about the biological component of the bio-psycho-social model on which modern pain care is expected to rest. This deficiency reasonably helps to explain the relatively small effects of treatment and rehabilitation. Our new cooperative multicentre project from all Swedish University hospitals combined will play a crucial part in the paradigm shift occurring and focusing on a personalised individual treatment method where we will be using advanced metabolomic as well as proteomic methods. The present project is an example of the paradigm shift that is now taking place in modern clinical medicine in a direction towards precision medicine where *omic* research is a crucial element. The new research is characterised by having a focus on biological processes and involves developing a clinical medicine, which is based not only on anamnesis and clinical examination but that also includes the possibility to measure the actual pathophysiological mechanisms at work in each patient (hence, precision medicine). This is crucial for the development of the clinical diagnosis and treatment of chronic pain and other conditions and diseases in which the clinically oriented researchers participating in this project are involved and engaged.

The project is on an international research front and has great potential to contribute to an improved understanding of activated nociceptive and pain mechanisms and thereby better diagnosis and, in the long term, treatment of chronic pain. It will contribute to the development of clinically useful saliva and blood samples that can be used in the investigation and choice of treatment for patients with chronic pain. In this way, the project will be able to contribute to the development of investigation and treatment that is indeed based on the individual patient’s activated nociception and pain mechanisms (‘personalised medicine’, ‘precision medicine’). The results will also be able to form the basis for new pharmacological development, helping to bridge the gap between clinical pain medicine and animal models (c.f. translation and backtranslation).

The feasibility of the project is very high. The research group has broad and adequately high competence for the implementation. The participating university clinics have working routines for collecting data for SQRP (response rate >90%) and routines for recruiting healthy participants. Extensive experience of the biochemical and statistical methods that will be used in the project is available. We have access to all necessary equipment partly within our research laboratory Painomics laboratory at Linköping University, through the Faculty of Medicine’s Core Facility at Linköping University and the local node in Linköping that is under construction and connected to the Swedish Nuclear Magnetic Resonance spectroscopy centre in Gothenburg.

In the studies that form the basis of the project and use the same methods, we have found marked differences in the protein/inflammation pattern in muscles and plasma at CWP/FM.^16 17 33^ In the proteomics studies of musculature and plasma, we have been able to identify proteins that with great certainty explain the group affiliation (patient or healthy) (R2: 0.81–0.84)^16 17^; similar results are obtained with the inflammation panel.^15^ The significant proteins in the proteomics studies reflect activated nociceptive, inflammatory and various metabolic processes. Similar conclusions are drawn in other existing proteomics studies.^17 43–45^ Our proteomics/inflammation research has received attention and been highlighted as innovative and internationally leading.^46 47^ A recently published systematic review identified that proteomics research on chronic pain is dominated by researchers from Sweden (mainly this research group).^48^

*Overall,* this clinically anchored project will be of great importance for the optimisation of the clinical investigation and assessment as well as for better and more adapted treatment interventions based on the activated nociceptive mechanisms. This project will fill an important gap in our knowledge of the molecular mechanisms of common chronic pain conditions in humans and will produce significant steps towards a mechanism based and precision pain medicine.

## Ethics

The study is approved by the Swedish Ethical Review Authority (Dnr 2021–04929) and will be conducted in accordance with the declaration of Helsinki. All participants will give their informed consent before the experiments and they will repeatedly be informed about their right to interrupt the participation at any time point, without explanation of their actions. Before data are registered in the database, it will be blinded. All measurements are performed by registered health care staff. The participants will receive written information with the telephone number of the project responsible physician to be contacted in case of complications. The visits, any complications and a summary of the study will be recorded in the electronic record. When taking samples and measuring pain sensitivity, participants may experience short periods of (increased) pain. However, this discomfort should not be more extensive than temporary pain that is experienced in everyday situations. The samples of blood and saliva will be frozen immediately after collection and stored in biobank at; Östergötland, reg nr 1, Örebro biobank, IVO reg nr 454, Biobank Norr, IVO reg nr 472, Uppsala Biobank, IVO reg nr 827, Biobank Väst, IVO reg nr 890, Region Skånes biobank, IVO reg nr 136 and the local biobank at Danderyd hospital AB in Stockholm.

Results will be presented at the group level without the possibility of identifying any single individual. Overall, our assessment is that the benefits of this project are considerably greater than the risks.

## Supplementary Material

Reviewer comments

Author's manuscript
