# Supplementary material for: Swedish Chronic Pain Biobank: protocol for a multicentre registry and biomarker project
Source: BMJ Open. 2022 Nov 30;12(11):e066834. doi: 10.1136/bmjopen-2022-066834 (PMC9717004; doi:10.1136/bmjopen-2022-066834)
Supplement: Supplementary data [file bmjopen-2022-066834supp001.pdf]

Appendix 1

Table: References to psychometric characteristics for the patient reported outcome measures (cf. Table 1) to be used in the project.

**Instrument/Scale**	**References**
Pain intensity (NRS)	1
Fibromyalgia 2016 criteria	2
Psychological distress (HADS)	3 4
Multidimensional Pain Inventory (MPI)	5-7
Pain catastrophizing (PCS)	8 9
Insomnia (ISI)	10 11
Pain coping (CPAQ8)	12-14
Fear of movement (TSK)	15-18
Health-related quality of life (RAND-36)	19-22
Health-related quality of life (EQ-5D^{TM})	23-26
Physical activity	27 28

NRS = Numeric Rating Scale, MPI = Multidimensional Pain Inventory, HADS = Hospital Anxiety and Depression Scale, PCS = Pain Catastrophizing Scale, ISI = insomnia Severity Index, CPAQ = Chronic Pain Acceptance Questionnaire, TSK = Tampa Scale for Kinesiophobia, RAND-36 = modernized version of Short-Form Health Survey-36.

References

1. Dworkin RH, Turk DC, Farrar JT, et al. Core outcome measures for chronic pain clinical trials: IMMPACT recommendations. *Pain* 2005;113(1-2):9-19. [published Online First: 2004/12/29]
2. Wolfe F, Clauw DJ, Fitzcharles MA, et al. 2016 Revisions to the 2010/2011 fibromyalgia diagnostic criteria. *Semin Arthritis Rheum* 2016;46(3):319-29. doi: 10.1016/j.semarthrit.2016.08.012 [published Online First: 2016/12/06]
3. Lisspers J, Nygren A, Söderman E. Hospital Anxiety and Depression Scale (HAD): some psychometric data for a Swedish sample. *Acta Psychiatrica Scandinavica,* 1997;96(4):281-6.
4. Zigmond AS, Snaith RP. The hospital anxiety and depression scale. *Acta Psychiatr Scand* 1983;67(6):361-70. [published Online First: 1983/06/01]
5. Bergstrom G, Jensen IB, Bodin L, et al. Reliability and factor structure of the Multidimensional Pain Inventory--Swedish Language Version (MPI-S). *Pain* 1998;75(1):101-10. [published Online First: 1998/04/16]
6. Kerns RD, Turk DC, Rudy TE. The West Haven-Yale Multidimensional Pain Inventory (WHYMPI). *Pain* 1985;23(4):345-56. [published Online First: 1985/12/01]
7. Turk D, Rudy T. Toward an empirically derived taxonomy pf chronic pain patients: integration of psychological assessment data. *J Consult Clin Psychol* 1988;56:233-8.
8. Quartana PJ, Campbell CM, Edwards RR. Pain catastrophizing: a critical review. *Expert Rev Neurother* 2009;9(5):745-58. doi: 10.1586/ern.09.34
9. Sullivan M, Bishop S, Pivik J. The Pain catastrophizing scale: development and validation. *Psychol Assess* 1995;7:524-32.

10. Dragioti E, Wiklund T, Alfoldi P, et al. The Swedish version of the Insomnia Severity Index: Factor structure analysis and psychometric properties in chronic pain patients. *Scand J Pain* 2015;9(1):22-27. doi: 10.1016/j.sjpain.2015.06.001 [published Online First: 2018/06/19]
11. Jansson M, Linton SJ. Cognitive-behavioral group therapy as an early intervention for insomnia: a randomized controlled trial. *J Occup Rehabil* 2005;15(2):177-90. doi: 10.1007/s10926-005-1217-9 [published Online First: 2005/04/23]
12. Rovner GS, Arestedt K, Gerdle B, et al. Psychometric properties of the 8-item Chronic Pain Acceptance Questionnaire (CPAQ-8) in a Swedish chronic pain cohort. *J Rehabil Med* 2014;46(1):73-80. doi: 10.2340/16501977-1227
13. Fish RA, McGuire B, Hogan M, et al. Validation of the chronic pain acceptance questionnaire (CPAQ) in an Internet sample and development and preliminary validation of the CPAQ-8. *Pain* 2010;149(3):435-43. doi: 10.1016/j.pain.2009.12.016 [published Online First: 2010/03/02]
14. Vowles KE, McCracken LM, McLeod C, et al. The Chronic Pain Acceptance Questionnaire: confirmatory factor analysis and identification of patient subgroups. *Pain* 2008;140(2):284-91. doi: 10.1016/j.pain.2008.08.012 [published Online First: 2008/10/01]
15. Lundberg M, J. S, SG. C. A psychometric evaluation of the Tampa Scale for Kinesiophobia - from a physiotherapeutic perspective. *Physiotherapy Theory and Practice* 2004;20(2):121 - 33
16. Cleland JA, Fritz JM, Childs JD. Psychometric properties of the Fear-Avoidance Beliefs Questionnaire and Tampa Scale of Kinesiophobia in patients with neck pain. *Am J Phys Med Rehabil* 2008;87(2):109-17. doi: 10.1097/PHM.0b013e31815b61f1
17. Miller R, Kori S, Todd D. The Tampa Scale: a measure of kinesiophobia. *Clin J Pain* 1991;7(1):51-52.
18. Roelofs J, Goubert L, Peters ML, et al. The Tampa Scale for Kinesiophobia: further examination of psychometric properties in patients with chronic low back pain and fibromyalgia. *Eur J Pain* 2004;8(5):495-502. doi: 10.1016/j.ejpain.2003.11.016
19. Sullivan M, Karlsson, J., Ware, J. The Swedish 36 Health survey. Evaluation of data quality, scaling assumption, reliability and construct validity across general populations in Sweden. *Soc Sci Med* 1995;41:1349-58.
20. Hays RD, Morales LS. The RAND-36 measure of health-related quality of life. *Ann Med* 2001;33(5):350-7. doi: 10.3109/07853890109002089 [published Online First: 2001/08/09]
21. Hays RD, Sherbourne CD, Mazel RM. The RAND 36-Item Health Survey 1.0. *Health Econ* 1993;2(3):217-27. doi: 10.1002/hec.4730020305 [published Online First: 1993/10/01]
22. Orwelius L, Nilsson M, Nilsson E, et al. The Swedish RAND-36 Health Survey - reliability and responsiveness assessed in patient populations using Svensson's method for paired ordinal data. *J Patient Rep Outcomes* 2017;2(1):4. doi: 10.1186/s41687-018-0030-0 [published Online First: 2017/01/01]
23. Karlsson JA, Nilsson JA, Neovius M, et al. National EQ-5D tariffs and quality-adjusted life-year estimation: comparison of UK, US and Danish utilities in south Swedish rheumatoid arthritis patients. *Ann Rheum Dis* 2011;70(12):2163-6. doi: 10.1136/ard.2011.153437 [published Online First: 2011/08/24]
24. Cheung K, Oemar M, Oppe M, et al. User Guide. Basic information on how to use EQ-5D 2009 [2.0:[Available from: www.eurogol.org accessed 20120101.
25. Brooks R. EuroQol: the current state of play. *Health Policy* 1996;37(1):53-72.
26. EuroQol. EuroQol: a new facility for the measurement of health-related quality of life. *Health Policy* 1990;16:199-208.
27. Kallings L. Validering av Socialstyrelsens screeningfrågor om fysisk aktivitet Stockholm: The Swedish School of Sport and Health Sciences; 2015 [Available from: www.socialstyrelsen.se accessed 20151202.
28. Olsson S. Studies of physical activity in the Swedish population. The Swedish School of Sport and Health Sciences, 2016.
