# Supplementary material for: Swedish Chronic Pain Biobank: protocol for a multicentre registry and biomarker project
Source: BMJ Open. 2022 Nov 30;12(11):e066834. doi: 10.1136/bmjopen-2022-066834 (PMC9717004; doi:10.1136/bmjopen-2022-066834)
Supplement: Author's manuscript [file bmjopen-2022-066834.draft_revisions.pdf]

BMJ Open

BMJ Open is committed to open peer review. As part of this commitment we make the peer review history of every article we publish publicly available.

When an article is published we post the peer reviewers' comments and the authors' responses online. We also post the versions of the paper that were used during peer review. These are the versions that the peer review comments apply to.

The versions of the paper that follow are the versions that were submitted during the peer review process. They are not the versions of record or the final published versions. They should not be cited or distributed as the published version of this manuscript.

BMJ Open is an open access journal and the full, final, typeset and author-corrected version of record of the manuscript is available on our site with no access controls, subscription charges or pay-per-view fees (http://bmjopen.bmj.com).

If you have any questions on BMJ Open's open peer review process please email info.bmjopen@bmj.com

BMJ Open

The Swedish chronic Pain Biobank - protocol for a multicenter registry and biomarker project

Journal:	*BMJ Open*
Manuscript ID	bmjopen-2022-066834
Article Type:	Protocol
Date Submitted by the Author:	25-Jul-2022
Complete List of Authors:	Ghafouri, B; Linköping University, Department of Health, Medicine and Caring Sciences<br>Ernberg, Malin; Karolinska Institute Department of Dental Medicine<br>Andre'll PhD, Paulin; Sahlgrenska Academy, Department of Molecular and Clinical Medicine/ Pain Centre, Institute of Medicine<br>Bäckryd, Emmanuel; Linköping University, Department of Health, Medicine and Caring Sciences<br>Fisher, Marcelo Rivano; Lund University, Department of Neurosurgery and Pain Rehabilitation at Skåne University Hospital and Faculty of Medicine Department of Clinical Sciences Malmö; Lund University, Department of Health Sciences<br>Freund-Levi, Yvonne; Örebro University School of Medical Sciences; Karolinska Institute Department of Clinical Science and Education Södersjukhuset<br>Grelz, Henrik; Lund University, Department of Neurosurgery and Pain Rehabilitation at Skåne University Hospital and Faculty of Medicine Department of Clinical Sciences Malmö<br>Gräbel, Olaf; Sahlgrenska University Hospital<br>Karlsten, Rolf; Uppsala University Hospital, Department of Surgical Sciences, Anaesthesiology and Intensive Care<br>Kosek, Eva; Karolinska Institute<br>Löfgren, Monika; Karolinska Institutet, Department of Clinical Sciences<br>Ringqvist, Åsa; Lund University, Department of Neurosurgery and Pain Rehabilitation at Skåne University Hospital and Faculty of Medicine Department of Clinical Sciences Malmö<br>Rudling, Karin; Orebro universitet, Department of rehabilitation medicine<br>Stålnacke, Britt-Marie; Umea Universitet, Department of Community Medicine and Rehabilitation, Rehabilitation Medicine<br>Sörlén, Niklas; Umea University, Department of Clinical Science, Neurosciences<br>Uhlin, Karin; Karolinska Institutet, Department of Clinical Sciences<br>Westergren, Hans; Lund University, Department of Neurosurgery and Pain Rehabilitation at Skåne University Hospital and Faculty of Medicine Department of Clinical Sciences Malmö; Lund University, Department of Health Sciences<br>Gerdle, Björn; Linkopings universitet, Pain and Rehabilitation Centre, and Department of Medical and Health Sciences
Keywords:	REHABILITATION MEDICINE, PAIN MANAGEMENT, Neurological pain < NEUROLOGY, Back pain < ORTHOPAEDIC & TRAUMA SURGERY

SCHOLARONE™
Manuscripts

The Swedish chronic Pain Biobank - protocol for a multicenter registry and biomarker project

Bijar Ghafouri[1], Malin Ernberg[2], Paulin Andréll[3,4], Emmanuel Bäckryd[1], Marcelo Rivano Fisher[5,6], Yvonne Freund-Levi[7,8,9], Henrik Grelz[5], Olaf Gräbel[3], Rolf Karlsten[10], Eva Kosek[11], Monika Löfgren[12], Åsa Ringqvist[5], Karin Rudling[13], Britt-Marie Stålnacke[12,14], Niklas Sörlén[15], Karin Uhlin[12], Hans Westergren[5,6], Björn Gerdle[1]

[1]Pain and Rehabilitation Centre, and Department of Health, Medicine and Caring Sciences, Linköping University, SE 581 83 Linköping, Sweden
[2]Department of Dental Medicine, Karolinska Institutet, and the Scandinavian Center for Orofacial Neurosciences (SCON), Box 4064, SE14104 Huddinge Sweden
[3]Region Västra Götaland, Sahlgrenska University Hospital, Östra, department of Anaesthesiology and Intensive Care Medicine, Pain Centre, Box 426, SE 405 30 Gothenburg, Sweden
[4]Department of Anaesthesiology and Intensive Care Medicine, Institute of Clinical Sciences at Sahlgrenska Academy, University of Gothenburg, SE 416 85 Gothenburg, Sweden
[5]Department of Neurosurgery and Pain Rehabilitation at Skåne University Hospital and Faculty of Medicine Department of Clinical Sciences Malmö, Lund University, Jan Waldenströms gata 35, 202 13 Malmö, Sweden
[6]Rehabilitation Medicine Research Group, Department of Health Sciences, Lund University, Lund, Sweden
[7]School of Medical Sciences, Örebro University and department of Geriatrics University Hospital Örebro
[8]Department of geriatrics, Södertälje Hospital
[9]Department of Clinical Science and Education, Södersjukhuset, Karolinska Institutet, Stockholm
[10]Department of Surgical Sciences, Anaesthesiology and Intensive Care, Uppsala University Hospital, 751 85 Uppsala, Sweden
[11]Department Surgical Sciences, Uppsala University, Uppsala, and Department of Clinical Neuroscience, Karolinska Institutet, Stockholm, Sweden
[12]Department of Clinical Sciences, Karolinska Institutet, and Department of Rehabilitation Medicine Danderyd Hospital, SE 182 88 Stockholm Sweden
[13]Department of rehabilitation medicine, University hospital Örebro, Sweden
[14]Department of Community Medicine and Rehabilitation, Rehabilitation Medicine, Umeå University, SE 901 87 Umeå, Sweden
[15]Department of Clinical Science, Neurosciences, Umeå University, SE 901 87 Umeå, Sweden

Corresponding author
Bijar Ghafouri, Professor
Pain and Rehabilitation Medicine
Department of Health, Medicine, and Caring Sciences
Linköping University, SE-581 85 Linköping, Sweden
Tel.: +46-13-282664
E-mail address: Bijar.ghafouri@liu.se

Abbreviated title: Chronic pain biobank
Word count: 3672

For peer review only - http://bmjopen.bmj.com/site/about/guidelines.xhtml

Abstract

Introduction: About 20% of the adult population has chronic pain, often associated with psychological distress, sick leave, and poor health. There are large variations in the clinical picture. A biopsychosocial approach is used in investigation and treatment. The concept of personalized medicine, i.e., optimizing medication types and dosages for individual patients based on biomarkers and other patient-related factors, has received increasing attention in different diseases but used less in chronic pain. This cooperative project from all Swedish Universities investigates whether there are changes in protein, inflammation and metabolism patterns in saliva and blood in chronic pain patients and whether the changes correlate with clinical characteristics and rehabilitation outcomes.

Methods and analysis: Patients at multidisciplinary pain centers at University Hospitals in Sweden who have chosen to participate in the Swedish Quality Registry for Pain Rehabilitation and healthy gender- and age-matched individuals will be included in the project. Saliva and plasma samples will be collected in addition to questionnaire data obtained from the Register. From the samples, protein, inflammation, and metabolism patterns are analyzed in relation to, for example, diagnosis, pain characteristics, psychological distress, body weight, pharmacological treatment, and clinical rehabilitation results.

Ethics and Dissemination: The study is approved by the Swedish Ethical Review Authority (Dnr 2021-04929) and will be conducted in accordance with the declaration of Helsinki. The results will be published in open access scientific journals and in popular scientific relevant journals such as those from patient organizations. Data will be also presented in scientific meetings, meeting with healthcare organization and disseminated in different lecturers at the clinic and university.

Keywords: Biomarkers, Chronic pain, Inflammation, Metabolomics, Molecular profile, Proteomics

Strengths and limitations of this study

- This project will play a crucial part in the paradigm shift occurring and focusing on a personalized individual treatment method in chronic pain.

For peer review only - http://bmjopen.bmj.com/site/about/guidelines.xhtml

- The research group has broad and adequately high competence for the implementation.
- The participating university clinics have working routines for collecting data and routines for recruiting participants.
- Extensive experience of the clinical, biochemical, and statistical research is available in the research group.
- There is a risk of heterogeneity of the samples as this study is a multicenter study in a large geographical area where the infrastructure for data collection is different.
- Individuals not able to read and write Swedish are excluded which make the results less generalizable to the whole population.

For peer review only

For peer review only - http://bmjopen.bmj.com/site/about/guidelines.xhtml

1. Introduction

About 20% of the adult population lives with at least moderate-severe chronic pain; often with concomitant complex psychological distress including depression and anxiety. Pain will also reduce patients' ability to study or work full time, they will show higher levels of sick leave, and show reduced quality of life [2]. There are large variations in the clinical picture in chronic pain patients e.g., regarding psychiatric comorbidities; about 40% of patients managed at specialist level have co-morbid symptoms of depression and/or anxiety [3]. More than 20% of Years lived with disabilities – a measure of non-fatal disabilities - are caused by of pain conditions both in Sweden and globally [38].

Acute tissue damage initiates plastic and reinforcing mechanisms in the pain system peripherally and centrally, which in interaction with psychological and social factors create the experience of pain at a given moment. Chronic pain and in parallel depression constitute a large part of the pain complex, why depression also must be considered when diagnosing the patient. Depression in turn intensifies the pain by pain facilitating pathways. Social factors such as working conditions can be risk factors for the chronification of pain, and when chronic pain has developed return to work will be more difficult (i.e., chronic pain has social consequences). Modern pain care is therefore based on a bio-psycho-social approach to diagnosis and treatment [9; 12].

Chronic pain is not an acute pain that persists over time, but further plastic neurobiological changes occur in interaction with psychological and social factors. Imaging techniques have provided an in-depth understanding of how the brain processes and creates the experience of pain. Chronic pain is associated with chemical changes in structures in the brain that process pain, changes in the physiological interactions between these structures and in the descending control of nociception, and central hyperexcitability (central sensitization) [7; 23].

Chronic widespread pain (CWP) including fibromyalgia (FM) is the extreme of complex pain condition. FM is characterized by altered nociception and is a prototype of a nociplastic pain condition, however, it is well known that patients initially suffering from nociceptive pain can in time develop nociplastic pain conditions [22]. It has been argued that CWP / FM is a typical example of a central pain condition, i.e., peripheral factors

For peer review only - http://bmjopen.bmj.com/site/about/guidelines.xhtml

For peer review only

have little or no role. Others believe that peripheral factors initiate and perpetuate the central changes e.g., which is the case in coxarthrosis where central changes are normalized after hip replacement [32].

In various studies, we have shown an increased presence of pain-mediating (e.g., glutamate, serotonin), metabolic (e.g., lactate and pyruvate) and analgesic (e.g., N-acylethanolamines (NAE)) substances in muscle in neck-shoulder pain and in CWP / FM [16]. Other researchers have recently shown changes in the peripheral nociceptors of FM [44]. Proteomic studies are increasingly being performed in the field of pain medicine. Using targeted and untargeted proteomics, our research group has found significant differences in the protein/inflammation pattern in muscles and plasma in CWP / FM [17; 26; 42]. Such changes also correlate with, for example, pain intensity, pain sensitivity and psychological strain [17; 27]. Taken together, this provides support for peripheral factors that significantly contribute to the maintenance of CWP / FM.

In the literature it is suggested that low-grade peripheral or systemic inflammation perpetuates chronic pain and is also involved in psychological / psychiatric conditions such as depression and obesity [11; 39]. Various pro-inflammatory cytokines and chemokines have been studied. However, the literature is not consistent; in a large FM study, we found no changes in “classic” pro-inflammatory cytokines in muscle or plasma [10]. Two recent systematic reviews of peripheral cytokines and chemokines - mainly based on single or few molecules - have not been consistent [1; 25]. Such hypothesis-driven studies have generally focused on a few molecules, while there are only a few exploratory studies of a larger number of molecules (panels) is few. Low-grade inflammation/neuroinflammation has also been studied in neuropathic pain conditions [5; 6; 19].

Preliminary biomarker data available in the literature need to be confirmed in larger studies that reflect the normal flow of chronic pain patients where/in which comparisons are made between pain diagnoses and that include control for gender, pain characteristics, co-morbidity, body weight, and pharmacological therapy. The inflammation pattern according to “classic” inflammation markers seems to be of preliminary importance for the treatment outcome in depression [30] and obesity, as well as for psychological treatment, such as cognitive behavioral therapy (CBT) in chronic

For peer review only - http://bmjopen.bmj.com/site/about/guidelines.xhtml

pain [24]. Physical exercise provides anti-inflammatory effects [21; 36]. Interdisciplinary Pain Rehabilitation Programs (IPRP), in which psychological treatment (including CBT) and physical activity and exercise are important components, constitute evidence-based treatment for chronic pain but with small to moderate effect sizes [4; 20; 28; 33]. It has not yet been considered if activated biological mechanisms may impact on the effect of IPRP. It is therefore important to investigate whether IPRP result is related to protein, inflammation, and metabolism patterns.

Aim and hypotheses

The aim of this multicenter project is to investigate whether there are differences in protein, inflammation and metabolism patterns between common chronic pain diagnoses and age- and gender-matched healthy controls from the general Swedish population. Further aims are to characterize and compare the changes of the levels of protein, inflammation, and metabolism in different diagnostic groups of chronic pain patients regarding e.g., gender, diagnosis, pain, pain sensitivity, co-morbidity pattern, body weight, pharmacological therapy, and clinical rehabilitation outcomes.

The hypotheses are:

A) there are unique biomarker signatures in plasma and saliva that distinguish common chronic pain diagnoses from each other and from healthy controls.

B) there are significant correlations between the identified biomarker profiles and pain characteristics e.g., intensity, pain sensitivity and anatomical spread of pain, co-morbidity patterns, body weight and pharmacological therapy, and clinical rehabilitation outcomes.

For peer review only

For peer review only - http://bmjopen.bmj.com/site/about/guidelines.xhtml

2. Methods

2.1. Participants

This multicenter project includes patients (> 18 years) with chronic pain who are recruited from the *Swedish Quality Registry for Pain Rehabilitation* (SQRP) and healthy gender- and age-matched controls from university hospital at Linkoping, Stockholm, Uppsala, Umea, Gothenburg, and Lund.

2.2. Patients with chronic pain

The project mainly uses patient-reported data from SQRP (www.ucr.uu.se/nrs/) for the participating university clinics. The project leader (Björn Gerdle) is the research leader for the national research group for SQRP and a member of its steering group.

SQRP is based on *Patient Reported Outcome Measures* (PROM) in the form of a written questionnaire. Validated instruments are used in the survey and collectively capture the various facets of the International Classification of Functional Conditions, Disabilities and Health (ICF). The survey includes background variables (*age, gender, country of origin, education and healthcare consumption, weight, and height*) and pain characteristics, degree of FM-ness, psychological distress, difficulty sleeping, pain coping, fear of movement, consequences of chronic pain concerning activity level and function, physical activity level, as well as health-related quality of life and perceived health (Table 1). The response rate is> 90% in SQRP, which can be interpreted as patients considering it important to be able to share how they experience their situation. The current diagnoses according to ICD-10 are also registered in SQRP.

Table 1. Patient reported outcome measures to be used in the project. NRS = Numeric Rating Scale, MPI = Multidimensional Pain Inventory, ACR = American College of Rheumatology, HADS = Hospital Anxiety and Depression Scale, PCS = Pain Catastrophizing Scale, ISI = insomnia Severity Index, CPAQ = Chronic Pain Acceptance Questionnaire, TSK = Tampa Scale for Kinesiophobia, RAND-36 = modernized version of Short-Form Health Survey-36.

**Survey**	**Instrument**	**Properties**
Pain characteristics		
Intensity	NRS (0-10), MPI part 1	0 = no pain, 10 = worst pain experienced
Anatomical extent	Pain drawing?	Body map front and back side
Frequency	Scale?	0 = never, 1 =, 2 =, 3 =?
Duration		Years
Fibromyalgia		
	ACR 2016 criteria	Widespread pain index (WPI): 0-19 sites

For peer review only - http://bmjopen.bmj.com/site/about/guidelines.xhtml

		Symptom severity score (SSS): 0-12<br>FMness score: WPI + SSS.
Psychological distress		
Depression	HADS (0-21)	7 items, each scored 0-3 according to frequency a summed
Anxiety	HADS (0-21)	7 items, each scored 0-3 according to frequency and summed
	MPI part 1 (0-6)	
	Life control?	2 items scored 0-6
	Affective distress?	3 items scored 0-6<br>The mean of each subscale is calculated
Pain catastrophizing	PCS (0-52)	13 items, each scored 0-4 according to frequency and summed
Difficulty sleeping		
	ISI (0-28)	7 items, each scored 0-4 according to frequency and summed
Pain coping		
	CPAQ (0-156?)	20 items, each scored 0-3 according to how often statement is true and summed
Fear of movement		
	TSK	17 items, each scored 0-4 according to agreement with statement and summed
Consequences chronic pain		
Activity level	MPI part 1 and 3 (0-6)	18 items each scored 0-6. The mean for each subscale or the total mean is calculated
Function	MPI part 1 and 3 (0-6)	20 items, each scored 0-6. The mean for each subscale or the total mean is calculated
Health-related quality of life		
	RAND-36 (0-100)	36 items in 8 subcategories scored varying between 0 and 100% (with fixed intervals). The mean for each subscale is calculated
	EQ-5D™	Consists of two parts. The first part is an index obtained from five dimensions items scored 0-5. The second part is self-estimation of today's health according to a 100-point thermometer-like scale (EQ5D-VAS) with defined end points (high values indicate better health and low values indicate worse health)
Physical activity		
Sedentary behavior		
Low intensity		Minutes per week
Moderate intensity		
Vigorous intensity		

The patients who are referred to the clinics respond to the SQRP questionnaire in connection with clinical assessment. Patients participating in IPRP also answer questionnaires immediately before, after and 12 months after rehabilitation.

All patients who respond to SQRP at the relevant university clinics are asked to participate in the project and may submit saliva samples and blood samples (see below).

For peer review only

For peer review only - http://bmjopen.bmj.com/site/about/guidelines.xhtml

In this project we will study the following 11 conditions/diagnoses (based on the manual produced within SQRP by, among others, the project leader):

1. Chronic neck-shoulder pain (cervicalgia, trapezius myalgia)
2. Chronic low back pain without sciatica
3. Generalized pain (CWP) according to criteria ACR 2016 criteria.
4. Fibromyalgia according to the ACR 2016 criteria.
5. Neuropathic pain including sciatica
6. Chronic Whiplash Disorders (WAD)
7. Ehlers-Danlos Syndrome (EDS) or Hypermobility Syndrome (HMS)
8. Complex Regional Pain Syndrome (CRPS)
9. Pain post-COVID infection
10. Chronic pain, not elsewhere classified (ICD R52.1, R52.2 and R52.9)

In addition, we will investigate the patterns when several pain diagnoses are present simultaneously.

2.3. Healthy controls

Age- and gender-matched healthy control persons are recruited via advertisements and answer a customized version of the SQRP questionnaire. They donate saliva samples and blood samples. Half of the healthy controls are asked to submit new samples after about 10 weeks.

2.4. Exclusion criteria for patients and controls

Coagulation disorders with predisposition to bleeding, medication with anticoagulants (low-dose aspirin is permitted), hypersensitivity to anesthetic, serious psychiatric disease (investigator's judgement, e.g., psychosis or suicidal ideation), and difficulties in understanding the Swedish language.

2.5. Supplementary survey

In addition to the instruments included in the SQRP (or equivalent adapted for healthy controls), the participants also answer a brief questionnaire in close connection with the tissue sampling. This charts the time of sampling, fasting or not, current medication and health food preparations (e.g., curcumin), pain intensity, and present and previous illnesses.

For peer review only - http://bmjopen.bmj.com/site/about/guidelines.xhtml

2.6. Biofluids sampling

Sampling consists of saliva and blood samples (plasma) drawn in the morning and during fasting. Saliva samples are taken with Salivette® (Sarstedt AG & Co, PO Box 1220, D-51582, Nümbrecht, Germany, obtained from VWR, article number: 101093–968). The participants avoid brushing teeth or drinking (water is ok) at least one hour before sampling. They rinse their mouth with water and wait for 15 minutes before placing the swab in the mouth. After 3 minutes, they spit the swab into the salivette tube containing a protease cocktail inhibitor. The salivette will be centrifuged (2 minutes, 1,000 x g) and the supernatant is transferred to a new tube and aliquoted into 200 µl in 0.6 ml eppendorf tubes and stored at -86 ° C until analysis. The total volume of saliva will be recorded.

Blood samples (2 x 8.5 ml) are collected in P100 tubes (article number: 366448, BD Diagnostics System, Frankling Lakes, NJ, USA) according to the manufacture's recommendations. The sample is centrifuged at 2500 g for 20 minutes at room temperature within 2-4 hours. Plasma will be extracted by carefully removing the upper part of the supernatant about 2/3 in fractions to a 10 ml tube on ice. The plasma samples will be aliquoted into 200 µl in 0.6 ml eppendorf tubes and stored at -80 ° C until analysis .

The samples are handled according to previously developed methodology [26; 42].

2.7. Biochemical analyses

The analyzes that will be used in this study are mainly exploratory, i.e., which proteins, metabolites and lipoproteins will be identified cannot be determined in advance. The following analyzes will be performed according to the methodology previously described by us [17; 19; 42].

- Exploratory analyzes using panels for inflammation, cytokines & chemokines and neuroinflammation comprising many proteins (from Olink Bioscience, Uppsala [17] and from Meso Scale Discovery [19].
- Exploratory proteomics analysis using 2D gel electrophoresis and/or shotgun proteomics [26; 43]
- Exploratory Metabolomic and Lipoprotein profile including free fatty acids (FFAs)

For peer review only - http://bmjopen.bmj.com/site/about/guidelines.xhtml

- Anti-nociceptive substances, i.e., endocannabinoids, NAE, endorphins, alpha-amylase, and cortisol.
- Metabolites such as lactate, pyruvate, glutamate, and other single pain-mediating molecules such as substance P, bradykinin, serotonin and BDNF.
- Pending analysis, the samples are stored in the biobank facilities with which the participating university clinics are associated.

2.8. Data from SQRP

The surveys in SQRP, including the supplementary questionnaire, will include a careful characterization of the patients and the healthy controls. In the analyzes against the saliva and blood samples, the following variables will initially be used:

- Diagnosis (from manual produced within SQRP)
- Pain intensity
- Widespreadness of pain/Pain localization
- Pain duration
- Psychologic distress (depression, anxiety, pain catastrophizing, fear of movement and insomnia)
- Sex
- Body mass index (BMI)
- Physical activity level
- Pharmacological therapy and health food preparations

2.9. Interdisciplinary multi modal rehabilitation program (IPRP)

The inclusion criteria are decided by the clinical examination and screening questionnaires concerning both anthropometric measures as well as subjective health followed by an inter professional conference between the MD, a psychologist, a physiotherapist, and an occupational therapist – choosing which patients they believe would benefit from the IPRP, in accordance with recommendations from the Swedish Agency for Health Technology Assessments and Assessment of Social Services. The patient should be motivated and have the potential for an active change to be included in IPRP. All patients will participate in a specific individual exercise with a physiotherapist (with both relaxation therapy and strengthening specific regions of the body to unburden), individual- and group therapy led

For peer review only - http://bmjopen.bmj.com/site/about/guidelines.xhtml

by a clinical psychologist (cognitive behavioral therapy, education, coping and mindfulness) as well as environmental work changes from an occupational therapist, working with education and return to work strategies [34] in group of six to nine persons, commonly 20 h per week of group-based activities for 6 to 8 weeks [14]. Pain education (including lectures in basic pain science and pain management) will be offered both for patients as well as for their relatives, friends, and colleagues.

2.10. Patient and Public Involvement

Patients and the public were not involved in this research study.

2.11. Statistics

In addition to descriptive statistics, advanced multivariate statistics (MVDA [18]; i.e., advanced principal component analysis (PCA) and OPLS regressions) will be used according to the guidelines presented by Wheelock & Wheelock for omics data [40] using SIMCA®-P + (version17 .0, Sartorius AG, Göttingen Germany). The methods are necessary to manage and take advantage of the intercorrelation pattern (i.e., multicollinearity) between the identified proteins/substances. MVDA enables analyzes where the number of variables is significantly greater than the number of observations (i.e., short and broad data tables), which more traditional multivariate regressions cannot easily handle. The research group has a long experience of the methods and has used these in previous studies and has an established collaboration with statisticians.

2.12 Sample size calculation

To our knowledge, there are no methods for determining the sample size at MVDA. In the literature, for example, proteomics and metabolomics studies for chronic pain have so far been rather small and usually include 20 + 20 participants. The literature points out the need for large cohorts, especially in complex disease states such as chronic pain, and that the results are replicated in new cohorts. Here we make the assessment that at least 200 patients (from both sexes) in each of the 10 diagnostic groups (see above) must be recruited and the same number for replication of the results. In the group with several diagnoses, the heterogeneity is significant, so double the number is deemed necessary. The numbers are required to get a sufficient spread in terms of, for example, psychological load, BMI, and pharmacotherapy and to be able to identify clinically relevant subgroups within a diagnosis. Four hundred healthy controls (even gender

For peer review only - http://bmjopen.bmj.com/site/about/guidelines.xhtml

distribution) are deemed necessary; half of these leave new samples after about 10 weeks. For analyzes of the relationships with treatment results (mixed diagnoses), 300 patients are considered necessary.

In summary, we intend to recruit a total of 400 patients in each diagnostic group (1-10) and 800 in mixed diagnostic group for analysis at one time, 300 patients participating in treatment leave samples immediately before the treatment period and are followed up on two occasions, and 400 healthy controls. This means that 4,800 patients and 400 healthy controls, i.e., a total of 5,200 participants, will be involved.

3. Discussion

Sweden has a population of about 10 million people, and the societal costs of approximately 20% of the adult population having moderate-severe chronic pain have been calculated by the Swedish Agency for Health Technology Assessments and Assessment of Social Services in 2003 as SEK 87 billion per year, corresponding to approximatively 1 billion US dollars per million inhabitants and year. Chronic pain is often associated with extensive suffering and poor health. At the same time, it is necessary to state that the effects of, for example, pharmacological treatment are limited; a maximum of 25–30% of the patients report significant and clinically valuable effects. The effect sizes for IPRP are only small to moderate according both to systematic reviews and to registry studies of real-world patients [8; 31].

The importance of developing mechanism-based diagnostics and treatment for chronic pain has been emphasized [37]. Pain clinicians largely lack precision medicine tools that can provide support for treatment choice. This constitutes a significant lack of knowledge about the biological component of the bio-psycho-social model on which modern pain care is expected to rest. This deficiency reasonably helps to explain the relatively small effects of treatment and rehabilitation. Our new cooperative multicenter project from all Swedish Universities combined will play a crucial part in the paradigm shift occurring and focusing on a personalized individual treatment method where we will be using advanced metabolomic as well as proteomic methods. The present project is an example of the paradigm shift that is now taking place in modern clinical medicine in a direction towards precision medicine where *omic* research is a crucial element. The

For peer review only - http://bmjopen.bmj.com/site/about/guidelines.xhtml

new research is characterized by having a focus on biological processes and involves developing a clinical medicine which is based not only on anamnesis and clinical examination but that also includes the possibility to measure the actual pathophysiological mechanism at work in each patient (hence, precision medicine). This is crucial for the development of the clinical diagnosis and treatment of chronic pain and other conditions and diseases in which the clinically oriented researchers participating in this project are involved and engaged.

The project is on an international research front and has great potential to contribute to an improved understanding of activated nociceptive and pain mechanisms and thereby better diagnosis and, in the long term, treatment of chronic pain. It will contribute to the development of clinically useful saliva and blood samples that can be used in the investigation and choice of treatment for patients with chronic pain. In this way, the project will be able to contribute to the development of investigation and treatment that is indeed based on the individual patient's activated nociception and pain mechanisms ("personalized medicine", "precision medicine"). The results will also be able to form the basis for new pharmacological development, helping to bridge the gap between clinical pain medicine and animal models (c.f. translation and back-translation).

The feasibility of the project is very high. The research group has broad and adequately high competence for the implementation. The participating university clinics have working routines for collecting data for SQRP; (response rate> 90%) and routines for recruiting healthy participants. Extensive experience of the biochemical and statistical methods that will be used in the project is available. We have access to all necessary equipment partly within our research laboratory Painomics laboratory at Linköping University, through the Faculty of Medicine's Core Facility at Linköping University and the local node in Linköping that is under construction and connected to the Swedish Nuclear Magnetic Resonance spectroscopy (NMR) center in Gothenburg.

In the studies that form the basis of the project and use the same methods, we have found marked differences in the protein/inflammation pattern in muscles and plasma at CWP/FM [26; 42; 43]. In the proteomics studies of musculature and plasma, we have been able to identify proteins that can with great certainty explain the group affiliation (patient or healthy) ($R2$: 0.81- 0.84) [26; 42]; similar results are obtained with the

For peer review only - http://bmjopen.bmj.com/site/about/guidelines.xhtml

inflammation panel [17]. The significant proteins in the proteomics studies reflect activated nociceptive, inflammatory, and various metabolic processes. Similar conclusions are drawn in other existing proteomics studies [18; 29; 41; 42]. Our proteomics/inflammation research has received attention and been highlighted as innovative and internationally leading [13; 35]. A recently published systematic review identified that proteomics research on chronic pain is dominated by researchers from Sweden (mainly this research group) [15].

Overall, this clinically anchored project will be of great importance for the optimization of the clinical investigation and assessment as well as for better and more adapted treatment interventions based on the activated nociceptive mechanisms. This project will fill an important gap in our knowledge of the molecular mechanisms of common chronic pain conditions in humans and will produce significant steps towards a mechanism based and precision pain medicine.

Ethics

The study is approved by the Swedish Ethical Review Authority (Dnr 2021-04929) and will be conducted in accordance with the declaration of Helsinki. All participants will give their informed consent before the experiments and they will repeatedly be informed about their right to interrupt the participation at any time point, without explanation of their actions. Before data are registered in the database it will be blinded. All measurements are performed by registered health care staff. The participants will receive written information with the telephone number of the project responsible physician to be contacted in case of complications. The visits, any complications, and a summary of the study will be recorded in the electronic record. When taking samples and measuring pain sensitivity, participants may experience short periods of (increased) pain. However, this discomfort should not be more extensive than temporary pain that is experienced in everyday situations.

The samples of blood and saliva will be frozen immediately after collection and stored in biobank at; Östergötland, reg nr 1, Örebro biobank, IVO reg nr 454, Biobank Norr, IVO reg nr 472, Uppsala Biobank, IVO reg nr 827, Biobank Väst, IVO reg nr 890, Region Skånes biobank, IVO reg nr 136 and the local biobank at Danderyd hospital AB in Stockholm.

For peer review only - http://bmjopen.bmj.com/site/about/guidelines.xhtml

Results will be presented at the group level without the possibility of identifying any single individual. *Overall*, our assessment is that the benefits of this project are considerably greater than the risks.

Authors' contributions

The original scientific idea: BGh, ME, EB, and BG. All authors have contributed to the study design, data collection, analysis methods and writing the protocol.

Competing interests

The authors declare that they have no competing interests

Funding

This work is supported by grants from the Petrus and Augusta Hedlunds Foundation (grant N/A), the Gun and Bertil Stohnes Foundation (grant N/A), the Loo and Hans Osterman Foundation (grant N/A), the Demens foundation (grant N/A), the Brain foundation (grant FO2018-0315), the Senil demens Särfond foundation Region Örebro (grant N/A), the Gamla Tjänarinnor foundation (grant N/A), the Swedish Research Council (grant 2018-02470) and the Research-ALF region Ostergotland (grant RÖ-834951).

References

[1] Andrés-Rodríguez L, Borràs X, Feliu-Soler A, Pérez-Aranda A, Angarita-Osorio N, Moreno-Peral P, Montero-Marin J, García-Campayo J, Carvalho AF, Maes M, Luciano JV. Peripheral immune aberrations in fibromyalgia: A systematic review, meta-analysis and meta-regression. Brain Behav Immun 2020;87:881-889.

[2] Breivik H, Collett B, Ventafridda V, Cohen R, Gallacher D. Survey of chronic pain in Europe: prevalence, impact on daily life, and treatment. Eur J Pain 2006;10(4):287-333.

[3] Bromley Milton M, Börsbo B, Rovner G, Lundgren-Nilsson A, Stibrant-Sunnerhagen K, Gerdle B. Is Pain Intensity Really That Important to Assess in Chronic Pain Patients? A Study Based on the Swedish Quality Registry for Pain Rehabilitation (SQRP). PLoS One 2013;8(6):e65483.

[4] Bujak BK, Regan E, Beattie PF, Harrington S. The effectiveness of interdisciplinary intensive outpatient programs in a population with diverse chronic pain conditions: a systematic review and meta-analysis. Pain Manag 2019;9(4):417-429.

[5] Bäckryd E, Lind AL, Thulin M, Larsson A, Gerdle B, Gordh T. High levels of cerebrospinal fluid chemokines point to the presence of neuroinflammation in peripheral neuropathic pain: a cross-sectional study of 2 cohorts of patients compared with healthy controls. Pain 2017;158(12):2487-2495.

[6] Bäckryd E, Themistocleous A, Larsson A, Gordh T, Rice ASC, Tesfaye S, Bennett DL, Gerdle B. Hepatocyte growth factor, colony-stimulating factor 1, CD40, and 11 other inflammation-

For peer review only - http://bmjopen.bmj.com/site/about/guidelines.xhtml

related proteins are associated with pain in diabetic neuropathy: exploration and replication serum data from the Pain in Neuropathy Study. Pain 2021.
[7] Cleve M, Gussew A, Wagner G, Bär KJ, Reichenbach JR. Assessment of intra- and inter-regional interrelations between GABA+, Glx and BOLD during pain perception in the human brain - A combined (1)H fMRS and fMRI study. Neuroscience 2017;365:125-136.
[8] Elbers S, Wittink H, Konings S, Kaiser U, Kleijnen J, Pool J, Köke A, Smeets R. Longitudinal outcome evaluations of Interdisciplinary Multimodal Pain Treatment programmes for patients with chronic primary musculoskeletal pain: A systematic review and meta-analysis. Eur J Pain 2022;26(2):310-335.
[9] Engel GL. The need for a new medical model: a challenge for biomedicine. Science 1977;196(4286):129-136.
[10] Ernberg M, Christidis N, Ghafouri B, Bileviciute-Ljungar I, Löfgren M, Larsson A, Palstam A, Bjersing J, Mannerkorpi K, Kosek E, Gerdle B. Effects of 15 weeks of resistance exercise on pro-inflammatory cytokine levels in the vastus lateralis muscle of patients with fibromyalgia. Arthritis Res Ther 2016;18(1):137.
[11] Felger JC, Lotrich FE. Inflammatory cytokines in depression: neurobiological mechanisms and therapeutic implications. Neuroscience 2013;246:199-229.
[12] Gatchel RJ, Peng YB, Peters ML, Fuchs PN, Turk DC. The biopsychosocial approach to chronic pain: scientific advances and future directions. Psychol Bull 2007;133(4):581-624.
[13] Gazerani P, Vinterhøj HSH. 'Omics': An emerging field in pain research and management. Future Neurology 2016;11(4):255-265.
[14] Gerdle B, Bäckryd E, Falkenberg T, Lundström E, Ghafouri B. Changes in inflammatory plasma proteins from patients with chronic pain associated with treatment in an interdisciplinary multimodal rehabilitation program - an explorative multivariate pilot study. Scand J Pain 2019;20(1):125-138.
[15] Gerdle B, Ghafouri B. Proteomic studies of common chronic pain conditions - a systematic review and associated network analyses. Expert Rev Proteomics 2020;17(6):483-505.
[16] Gerdle B, Ghafouri B, Ernberg M, Larsson B. Chronic musculoskeletal pain: review of mechanisms and biochemical biomarkers as assessed by the microdialysis technique. J Pain Res 2014;7:313-326.
[17] Gerdle B, Ghafouri B, Ghafouri N, Bäckryd E, Gordh T. Signs of ongoing inflammation in female patients with chronic widespread pain: A multivariate, explorative, cross-sectional study of blood samples. Medicine (Baltimore) 2017;96(9):e6130.
[18] Han C-L, Sheng Y-C, Wang S-Y, Chen Y-H, Kang J-H. Serum proteome profiles revealed dysregulated proteins and mechanisms associated with fibromyalgia syndrome in women. Scientific Reports 2020;10(1):12347.
[19] Jönsson M, Gerdle B, Ghafouri B, Bäckryd E. The inflammatory profile of cerebrospinal fluid, plasma, and saliva from patients with severe neuropathic pain and healthy controls-a pilot study. BMC Neurosci 2021;22(1):6.
[20] Kamper SJ, Apeldoorn AT, Chiarotto A, Smeets RJ, Ostelo RW, Guzman J, van Tulder MW. Multidisciplinary biopsychosocial rehabilitation for chronic low back pain. Cochrane Database Syst Rev 2014(9):Cd000963.
[21] Kawi J, Lukkahatai N, Inouye J, Thomason D, Connelly K. Effects of Exercise on Select Biomarkers and Associated Outcomes in Chronic Pain Conditions: Systematic Review. Biol Res Nurs 2016;18(2):147-159.
[22] Kosek E, Clauw D, Nijs J, Baron R, Gilron I, Harris RE, Mico JA, Rice ASC, Sterling M. Chronic nociplastic pain affecting the musculoskeletal system: clinical criteria and grading system. Pain 2021;162(11):2629-2634.
[23] Lamichhane B, Jayasekera D, Jakes R, Ray WZ, Leuthardt EC, Hawasli AH. Functional Disruptions of the Brain in Low Back Pain: A Potential Imaging Biomarker of Functional Disability. Front Neurol 2021;12:669076.

For peer review only

[24] Lasselin J, Kemani MK, Kanstrup M, Olsson GL, Axelsson J, Andreasson A, Lekander M, Wicksell RK. Low-grade inflammation may moderate the effect of behavioral treatment for chronic pain in adults. J Behav Med 2016;39(5):916-924.
[25] O'Mahony LF, Srivastava A, Mehta P, Ciurtin C. Is fibromyalgia associated with a unique cytokine profile? A systematic review and meta-analysis. Rheumatology (Oxford) 2021;60(6):2602-2614.
[26] Olausson P, Gerdle B, Ghafouri N, Sjöström D, Blixt E, Ghafouri B. Protein alterations in women with chronic widespread pain--An explorative proteomic study of the trapezius muscle. Sci Rep 2015;5:11894.
[27] Olausson P, Ghafouri B, Ghafouri N, Gerdle B. Specific proteins of the trapezius muscle correlate with pain intensity and sensitivity - an explorative multivariate proteomic study of the trapezius muscle in women with chronic widespread pain. J Pain Res 2016;9:345-356.
[28] Papadopoulou D, Fassoulaki A, Tsoulas C, Siafaka I, Vadalouca A. A meta-analysis to determine the effect of pharmacological and non-pharmacological treatments on fibromyalgia symptoms comprising OMERACT-10 response criteria. Clin Rheumatol 2016;35(3):573-586.
[29] Ramírez-Tejero JA, Martínez-Lara E, Rus A, Camacho MV, Del Moral ML, Siles E. Insight into the biological pathways underlying fibromyalgia by a proteomic approach. J Proteomics 2018;186:47-55.
[30] Rethorst CD, Toups MS, Greer TL, Nakonezny PA, Carmody TJ, Grannemann BD, Huebinger RM, Barber RC, Trivedi MH. Pro-inflammatory cytokines as predictors of antidepressant effects of exercise in major depressive disorder. Mol Psychiatry 2013;18(10):1119-1124.
[31] Ringqvist Å, Dragioti E, Björk M, Larsson B, Gerdle B. Moderate and Stable Pain Reductions as a Result of Interdisciplinary Pain Rehabilitation-A Cohort Study from the Swedish Quality Registry for Pain Rehabilitation (SQRP). J Clin Med 2019;8(6).
[32] Rodriguez-Raecke R, Niemeier A, Ihle K, Ruether W, May A. Structural brain changes in chronic pain reflect probably neither damage nor atrophy. PLoS One 2013;8(2):e54475.
[33] Salathé CR, Melloh M, Crawford R, Scherrer S, Boos N, Elfering A. Treatment Efficacy, Clinical Utility, and Cost-Effectiveness of Multidisciplinary Biopsychosocial Rehabilitation Treatments for Persistent Low Back Pain: A Systematic Review. Global Spine J 2018;8(8):872-886.
[34] Scascighini L, Toma V, Dober-Spielmann S, Sprott H. Multidisciplinary treatment for chronic pain: a systematic review of interventions and outcomes. Rheumatology (Oxford) 2008;47(5):670-678.
[35] Sommer C. Exploring pain pathophysiology in patients. Science 2016;354(6312):588-592.
[36] Teixeira AA, Lira FS, Pimentel GD, Oliveira de Souza C, Batatinha H, Biondo LA, Yamashita AS, Junior EA, Neto JC. Aerobic Exercise Modulates the Free Fatty Acids and Inflammatory Response During Obesity and Cancer Cachexia. Crit Rev Eukaryot Gene Expr 2016;26(3):187-198.
[37] Vardeh D, Mannion RJ, Woolf CJ. Toward a Mechanism-Based Approach to Pain Diagnosis. J Pain 2016;17(9 Suppl):T50-69.
[38] Vos T, Flaxman AD, Naghavi M, Lozano R, Michaud C, Ezzati M, Shibuya K, Salomon JA, Abdalla S, Aboyans V, Abraham J, Ackerman I, Aggarwal R, Ahn SY, Ali MK, Alvarado M, Anderson HR, Anderson LM, Andrews KG, Atkinson C, Baddour LM, Bahalim AN, Barker-Collo S, Barrero LH, Bartels DH, Basáñez MG, Baxter A, Bell ML, Benjamin EJ, Bennett D, Bernabé E, Bhalla K, Bhandari B, Bikbov B, Bin Abdulhak A, Birbeck G, Black JA, Blencowe H, Blore JD, Blyth F, Bolliger I, Bonaventure A, Boufous S, Bourne R, Boussinesq M, Braithwaite T, Brayne C, Bridgett L, Brooker S, Brooks P, Brugha TS, Bryan-Hancock C, Bucello C, Buchbinder R, Buckle G, Budke CM, Burch M, Burney P, Burstein R, Calabria B, Campbell B, Canter CE, Carabin H, Carapetis J, Carmona L, Cella C, Charlson F, Chen H, Cheng AT, Chou D, Chugh SS, Coffeng LE, Colan SD, Colquhoun S, Colson KE, Condon J, Connor MD, Cooper LT, Corriere M, Cortinovis M, de Vaccaro KC, Couser W, Cowie BC, Criqui MH, Cross M, Dabhadkar KC, Dahiya M, Dahodwala N, Damsere-Derry J, Danaei G, Davis A, De Leo D, Degenhardt L, Dellavalle R, Delossantos A, Denenberg J, Derrett S, Des Jarlais DC, Dharmaratne SD, Dherani M, Diaz-

For peer review only - http://bmjopen.bmj.com/site/about/guidelines.xhtml

Torne C, Dolk H, Dorsey ER, Driscoll T, Duber H, Ebel B, Edmond K, Elbaz A, Ali SE, Erskine H, Erwin PJ, Espindola P, Ewoigbokhan SE, Farzadfar F, Feigin V, Felson DT, Ferrari A, Ferri CP, Fèvre EM, Finucane MM, Flaxman S, Flood L, Foreman K, Forouzanfar MH, Fowkes FG, Franklin R, Fransen M, Freeman MK, Gabbe BJ, Gabriel SE, Gakidou E, Ganatra HA, Garcia B, Gaspari F, Gillum RF, Gmel G, Gosselin R, Grainger R, Groeger J, Guillemin F, Gunnell D, Gupta R, Haagsma J, Hagan H, Halasa YA, Hall W, Haring D, Haro JM, Harrison JE, Havmoeller R, Hay RJ, Higashi H, Hill C, Hoen B, Hoffman H, Hotez PJ, Hoy D, Huang JJ, Ibeanusi SE, Jacobsen KH, James SL, Jarvis D, Jasrasaria R, Jayaraman S, Johns N, Jonas JB, Karthikeyan G, Kassebaum N, Kawakami N, Keren A, Khoo JP, King CH, Knowlton LM, Kobusingye O, Koranteng A, Krishnamurthi R, Lalloo R, Laslett LL, Lathlean T, Leasher JL, Lee YY, Leigh J, Lim SS, Limb E, Lin JK, Lipnick M, Lipshultz SE, Liu W, Loane M, Ohno SL, Lyons R, Ma J, Mabweijano J, MacIntyre MF, Malekzadeh R, Mallinger L, Manivannan S, Marcenes W, March L, Margolis DJ, Marks GB, Marks R, Matsumori A, Matzopoulos R, Mayosi BM, McAnulty JH, McDermott MM, McGill N, McGrath J, Medina-Mora ME, Meltzer M, Mensah GA, Merriman TR, Meyer AC, Miglioli V, Miller M, Miller TR, Mitchell PB, Mocumbi AO, Moffitt TE, Mokdad AA, Monasta L, Montico M, Moradi-Lakeh M, Moran A, Morawska L, Mori R, Murdoch ME, Mwaniki MK, Naidoo K, Nair MN, Naldi L, Narayan KM, Nelson PK, Nelson RG, Nevitt MC, Newton CR, Nolte S, Norman P, Norman R, O'Donnell M, O'Hanlon S, Olives C, Omer SB, Ortblad K, Osborne R, Ozgediz D, Page A, Pahari B, Pandian JD, Rivero AP, Patten SB, Pearce N, Padilla RP, Perez-Ruiz F, Perico N, Pesudovs K, Phillips D, Phillips MR, Pierce K, Pion S, Polanczyk GV, Polinder S, Pope CA, 3rd, Popova S, Porrini E, Pourmalek F, Prince M, Pullan RL, Ramaiah KD, Ranganathan D, Razavi H, Regan M, Rehm JT, Rein DB, Remuzzi G, Richardson K, Rivara FP, Roberts T, Robinson C, De Leòn FR, Ronfani L, Room R, Rosenfeld LC, Rushton L, Sacco RL, Saha S, Sampson U, Sanchez-Riera L, Sanman E, Schwebel DC, Scott JG, Segui-Gomez M, Shahraz S, Shepard DS, Shin H, Shivakoti R, Singh D, Singh GM, Singh JA, Singleton J, Sleet DA, Sliwa K, Smith E, Smith JL, Stapelberg NJ, Steer A, Steiner T, Stolk WA, Stovner LJ, Sudfeld C, Syed S, Tamburlini G, Tavakkoli M, Taylor HR, Taylor JA, Taylor WJ, Thomas B, Thomson WM, Thurston GD, Tleyjeh IM, Tonelli M, Towbin JA, Truelsen T, Tsilimbaris MK, Ubeda C, Undurraga EA, van der Werf MJ, van Os J, Vavilala MS, Venketasubramanian N, Wang M, Wang W, Watt K, Weatherall DJ, Weinstock MA, Weintraub R, Weisskopf MG, Weissman MM, White RA, Whiteford H, Wiersma ST, Wilkinson JD, Williams HC, Williams SR, Witt E, Wolfe F, Woolf AD, Wulf S, Yeh PH, Zaidi AK, Zheng ZJ, Zonies D, Lopez AD, Murray CJ, AlMazroa MA, Memish ZA. Years lived with disability (YLDs) for 1160 sequelae of 289 diseases and injuries 1990-2010: a systematic analysis for the Global Burden of Disease Study 2010. Lancet 2012;380(9859):2163-2196.

[39] Walker AK, Kavelaars A, Heijnen CJ, Dantzer R. Neuroinflammation and comorbidity of pain and depression. Pharmacol Rev 2014;66(1):80-101.

[40] Wheelock Å M, Wheelock CE. Trials and tribulations of 'omics data analysis: assessing quality of SIMCA-based multivariate models using examples from pulmonary medicine. Mol Biosyst 2013;9(11):2589-2596.

[41] Wåhlén K, Ernberg M, Kosek E, Mannerkorpi K, Gerdle B, Ghafouri B. Significant correlation between plasma proteome profile and pain intensity, sensitivity, and psychological distress in women with fibromyalgia. Scientific Reports 2020;10(1):12508.

[42] Wåhlén K, Olausson P, Carlsson A, Ghafouri N, Gerdle B, Ghafouri B. Systemic alterations in plasma proteins from women with chronic widespread pain compared to healthy controls: a proteomic study. J Pain Res 2017;10:797-809.

[43] Wåhlén K, Yan H, Welinder C, Ernberg M, Kosek E, Mannerkorpi K, Gerdle B, Ghafouri B. Proteomic Investigation in Plasma from Women with Fibromyalgia in Response to a 15-wk Resistance Exercise Intervention. Med Sci Sports Exerc 2022;54(2):232-246.

[44] Üçeyler N, Zeller D, Kahn AK, Kewenig S, Kittel-Schneider S, Schmid A, Casanova-Molla J, Reiners K, Sommer C. Small fibre pathology in patients with fibromyalgia syndrome. Brain 2013;136(Pt 6):1857-1867.

For peer review only - http://bmjopen.bmj.com/site/about/guidelines.xhtml

For peer review only

For peer review only - http://bmjopen.bmj.com/site/about/guidelines.xhtml

The Swedish Chronic Pain Biobank - protocol for a multicenter registry and biomarker project

Journal:	*BMJ Open*
Manuscript ID	bmjopen-2022-066834.R1
Article Type:	Protocol
Date Submitted by the Author:	15-Nov-2022

Complete List of Authors:	Ghafouri, B; Linköping University, Department of Health, Medicine and Caring Sciences<br>Ernberg, Malin; Karolinska Institute Department of Dental Medicine<br>Andre'll PhD, Paulin; Sahlgrenska Academy, Department of Molecular and Clinical Medicine/ Pain Centre, Institute of Medicine<br>Bäckryd, Emmanuel; Linköping University, Department of Health, Medicine and Caring Sciences<br>Fisher, Marcelo Rivano; Lund University, Department of Neurosurgery and Pain Rehabilitation at Skåne University Hospital and Faculty of Medicine Department of Clinical Sciences Malmö; Lund University, Department of Health Sciences<br>Freund-Levi, Yvonne; Örebro University School of Medical Sciences; Karolinska Institute Department of Clinical Science and Education Södersjukhuset<br>Grelz, Henrik; Lund University, Department of Neurosurgery and Pain Rehabilitation at Skåne University Hospital and Faculty of Medicine Department of Clinical Sciences Malmö<br>Gräbel, Olaf; Sahlgrenska University Hospital<br>Karlsten, Rolf; Uppsala University Hospital, Department of Surgical Sciences, Anaesthesiology and Intensive Care<br>Kosek, Eva; Karolinska Institute<br>Löfgren, Monika; Karolinska Institutet, Department of Clinical Sciences<br>Ringqvist, Åsa; Lund University, Department of Neurosurgery and Pain Rehabilitation at Skåne University Hospital and Faculty of Medicine Department of Clinical Sciences Malmö<br>Rudling, Karin; Orebro universitet, Department of rehabilitation medicine<br>Stålnacke, Britt-Marie; Umea Universitet, Department of Community Medicine and Rehabilitation, Rehabilitation Medicine<br>Sörlén, Niklas; Umea University, Department of Clinical Science, Neurosciences<br>Uhlin, Karin; Karolinska Institutet, Department of Clinical Sciences<br>Westergren, Hans; Lund University, Department of Neurosurgery and Pain Rehabilitation at Skåne University Hospital and Faculty of Medicine Department of Clinical Sciences Malmö; Lund University, Department of Health Sciences<br>Gerdle, Björn; Linkopings universitet, Pain and Rehabilitation Centre, and Department of Medical and Health Sciences

**Primary Subject Heading**:	Rehabilitation medicine

Secondary Subject Heading:	Public health
Keywords:	REHABILITATION MEDICINE, PAIN MANAGEMENT, Neurological pain < NEUROLOGY, Back pain < ORTHOPAEDIC & TRAUMA SURGERY

SCHOLARONE™
Manuscripts

The Swedish Chronic Pain Biobank - protocol for a multicenter registry and biomarker project

Bijar Ghafouri[1], Malin Ernberg[2], Paulin Andréll[3,4], Emmanuel Bäckryd[1], Marcelo Rivano Fisher[5,6], Yvonne Freund-Levi[7,8,9], Henrik Grelz[5], Olaf Gräbel[3], Rolf Karlsten[10], Eva Kosek[11], Monika Löfgren[12], Åsa Ringqvist[5], Karin Rudling[13], Britt-Marie Stålnacke[12,14], Niklas Sörlén[15], Karin Uhlin[12], Hans Westergren[5,6], Björn Gerdle[1]

[1]Pain and Rehabilitation Centre, and Department of Health, Medicine and Caring Sciences, Linköping University, SE 581 83 Linköping, Sweden
[2]Department of Dental Medicine, Karolinska Institutet, and the Scandinavian Center for Orofacial Neurosciences (SCON), Box 4064, SE14104 Huddinge Sweden
[3]Region Västra Götaland, Sahlgrenska University Hospital, Östra, department of Anaesthesiology and Intensive Care Medicine, Pain Centre, Box 426, SE 405 30 Gothenburg, Sweden
[4]Department of Anaesthesiology and Intensive Care Medicine, Institute of Clinical Sciences at Sahlgrenska Academy, University of Gothenburg, SE 416 85 Gothenburg, Sweden
[5]Department of Neurosurgery and Pain Rehabilitation at Skåne University Hospital and Faculty of Medicine Department of Clinical Sciences Malmö, Lund University, Jan Waldenströms gata 35, 202 13 Malmö, Sweden
[6]Rehabilitation Medicine Research Group, Department of Health Sciences, Lund University, Lund, Sweden
[7]School of Medical Sciences, Örebro University and department of Geriatrics University Hospital Örebro
[8]Department of geriatrics, Södertälje Hospital
[9]Department of Clinical Science and Education, Södersjukhuset, Karolinska Institutet, Stockholm
[10]Department of Surgical Sciences, Anaesthesiology and Intensive Care, Uppsala University Hospital, 751 85 Uppsala, Sweden
[11]Department Surgical Sciences, Uppsala University, Uppsala, and Department of Clinical Neuroscience, Karolinska Institutet, Stockholm, Sweden
[12]Department of Clinical Sciences, Karolinska Institutet, and Department of Rehabilitation Medicine Danderyd Hospital, SE 182 88 Stockholm Sweden
[13]Department of rehabilitation medicine, University hospital Örebro, Sweden
[14]Department of Community Medicine and Rehabilitation, Rehabilitation Medicine, Umeå University, SE 901 87 Umeå, Sweden
[15]Department of Clinical Science, Neurosciences, Umeå University, SE 901 87 Umeå, Sweden

Corresponding author
Bijar Ghafouri, Professor
Pain and Rehabilitation Medicine
Department of Health, Medicine, and Caring Sciences
Linköping University, SE-581 85 Linköping, Sweden
Tel.: +46-13-282664
E-mail address: Bijar.ghafouri@liu.se

Abbreviated title: Chronic pain biobank
Word count: 3672

For peer review only - http://bmjopen.bmj.com/site/about/guidelines.xhtml

Abstract

Introduction: About 20% of the adult population have chronic pain, often associated with psychological distress, sick leave, and poor health. There are large variations in the clinical picture. A biopsychosocial approach is used in investigation and treatment. The concept of personalized medicine, i.e., optimizing medication types and dosages for individual patients based on biomarkers and other patient-related factors, has received increasing attention in different diseases but used less in chronic pain. This cooperative project from all Swedish University Hospitals will investigate whether there are changes in inflammation and metabolism patterns in saliva and blood in chronic pain patients and whether the changes correlate with clinical characteristics and rehabilitation outcomes.

Methods and analysis: Patients at multidisciplinary pain centers at University Hospitals in Sweden who have chosen to participate in the Swedish Quality Registry for Pain Rehabilitation and healthy sex- and age-matched individuals will be included in the study. Saliva and blood samples will be collected in addition to questionnaire data obtained from the Register. From the samples, proteins, lipids, metabolites and micro-RNA will be analyzed in relation to, for example, diagnosis, pain characteristics, psychological distress, body weight, pharmacological treatment, and clinical rehabilitation results using advanced multivariate data analysis and bioinformatics.

Ethics and Dissemination: The study is approved by the Swedish Ethical Review Authority (Dnr 2021-04929) and will be conducted in accordance with the declaration of Helsinki. The results will be published in open access scientific journals and in popular scientific relevant journals such as those from patient organizations. Data will be also presented in scientific meetings, meeting with healthcare organization and disseminated in different lecturers at the clinic and university.

Keywords: Biomarkers, Chronic pain, Inflammation, Metabolomics, Molecular profile, Proteomics

Strengths and limitations of this study

For peer review only - http://bmjopen.bmj.com/site/about/guidelines.xhtml

- The major strength of this study is that both omics methods and patient reported outcomes measure from the Swedish Quality Registry for Pain Rehabilitation will be used.
- Researchers from different professions with strong research backgrounds in chronic pain, rehabilitation and omics collaborate in this pain precision medicine study.
- There is a risk of heterogeneity of the samples as this study is a multicenter study in a large geographical area where the infrastructure for data collection is different.
- Individuals not able to read and write Swedish are excluded which make the results less generalizable.

For peer review only

For peer review only - http://bmjopen.bmj.com/site/about/guidelines.xhtml

1. Introduction

About 20% of the adult population lives with at least moderate-severe chronic pain; often with concomitant complex psychological distress including depression and anxiety. Pain will also reduce patients' ability to study or work full time, they will show higher levels of sick leave, and show reduced quality of life [1]. There are large variations in the clinical picture in chronic pain patients because of the presence of different comorbidities. For example psychiatric comorbidities are common in chronic pain and about 40% of patients managed at specialist level have co-morbid symptoms of depression and/or anxiety [2]. More than 20% of Years lived with disabilities – a measure of non-fatal disabilities - are caused by of pain conditions both in Sweden and globally [3].

Acute tissue damage initiates plastic and reinforcing mechanisms in the pain system peripherally and centrally, which in interaction with psychological and social factors create the experience of pain at a given moment [4]. Chronic pain in combination with depression constitute a large part of the pain complex, why depression also must be considered when diagnosing the patient. Moreover, positive correlations exist between depressive symptoms and pain intensity. Social factors such as working conditions can be risk factors for the chronification of pain, and when chronic pain has developed return to work will be more difficult (i.e., chronic pain has social consequences). Modern pain care is therefore based on a bio-psycho-social approach to diagnosis and treatment[5, 6].

Chronic pain is not an acute pain that persists over time, but further plastic neurobiological changes occur in interaction with psychological and social factors. Imaging techniques have provided an in-depth understanding of how the brain processes and creates the experience of pain. Chronic pain is associated with chemical changes in I) structures in the brain that process pain, II) the physiological interactions between these structures, III) the descending control of nociception, and IV) central hyperexcitability (central sensitization)[7, 8].

Chronic widespread pain (CWP) including fibromyalgia (FM) is the extreme of complex pain condition. FM is characterized by altered nociception and is a prototype of a nociplasic pain condition, however, it is well known that patients initially suffering from

For peer review only

nociceptive pain can in time develop nociplastic pain conditions [9]. It has been argued that CWP / FM is a typical example of a central pain condition, i.e., that peripheral factors have little or no role. Others believe that peripheral factors initiate and perpetuate the central changes in analogy with e.g. coxarthrosis where central changes are normalized after hip replacement [10].

In various studies, we have shown an increased presence of pain-mediating (e.g., glutamate, serotonin), metabolic (e.g., lactate and pyruvate) and analgesic (e.g., N-acylethanolamines (NAE)) substances in muscle in neck-shoulder pain and in CWP / FM [11]. Other researchers have recently shown changes in the peripheral nociceptors of FM [12] and blood biomarkers in chronic pain [13, 14]. Proteomic studies are increasingly being performed in the field of pain medicine. Using targeted and untargeted proteomics, our research group has found significant differences in the protein/inflammation pattern in muscles and plasma in CWP / FM [15-17]. Such changes also correlate with, for example, pain intensity, pain sensitivity and psychological strain [15, 18]. Taken together, this provides support for peripheral factors that significantly contribute to the maintenance of CWP / FM.

In the literature it is suggested that low-grade peripheral or systemic inflammation perpetuates chronic pain and is also involved in psychological / psychiatric conditions such as depression and obesity [19, 20]. Various pro-inflammatory cytokines and chemokines have been studied. However, the literature is not consistent; in a large FM study, we found no changes in “classic” pro-inflammatory cytokines in muscle or plasma [21]. Two recent systematic reviews of peripheral cytokines and chemokines - mainly based on single or few molecules - have not been consistent [22, 23]. Such hypothesis-driven studies have generally focused on a few molecules, while there are only a few exploratory studies of a larger number of molecules (panels). Low-grade inflammation/neuroinflammation has also been studied in neuropathic pain conditions [24-26].

Preliminary biomarker data available in the literature need to be confirmed in larger studies that reflect the normal flow of chronic pain patients where/in which comparisons are made between pain diagnoses and that include control for gender, pain characteristics, co-morbidity, body weight, and pharmacological therapy. The

For peer review only - http://bmjopen.bmj.com/site/about/guidelines.xhtml

inflammation pattern according to “classic” inflammation markers seems to be of preliminary importance for the treatment outcome in depression [27] and obesity, as well as for psychological treatment, such as cognitive behavioral therapy (CBT) in chronic pain [28]. Interdisciplinary Pain Rehabilitation Programs (IPRP), in which psychological treatment (including CBT) and physical activity and exercise are important components, constitute evidence-based treatment for chronic pain but with small to moderate effect sizes [29-32]. It has not yet been considered if activated biological mechanisms may impact on the effect of IPRP. It is therefore important to investigate whether IPRP result is related to inflammation, and metabolism patterns. To the best of our knowledge there are no reports on established pain biobank that combine health informatics with biomarkers and bioinformatics to study chronic pain mechanisms in everyday life patients.

Aim and hypotheses

The aim of this multicenter project is to investigate whether there are differences in inflammation and metabolism patterns between common chronic pain diagnoses and to sex- and gender-matched healthy controls from the general Swedish population. Further aims are to characterize and compare the changes of the grade of inflammation, and metabolism in different diagnostic groups of chronic pain patients regarding e.g., sex, diagnosis, pain, pain sensitivity, co-morbidity pattern, body weight, pharmacological therapy, and clinical rehabilitation outcomes.

The hypotheses are:

A) there are unique biomarker signatures in plasma and saliva that distinguish common chronic pain diagnoses from each other and from healthy controls.

B) there are significant correlations between the identified biomarker profiles and pain characteristics e.g., intensity, pain sensitivity and anatomical spread of pain, co-morbidity patterns, body weight and pharmacological therapy, and clinical rehabilitation outcomes.

For peer review only

For peer review only - http://bmjopen.bmj.com/site/about/guidelines.xhtml

2. Methods

2.1. Participants

This multicenter project will include patients (> 18 years) with chronic pain who are recruited from the *Swedish Quality Registry for Pain Rehabilitation* (SQRP) and healthy sex- and age-matched controls from university hospital at Linkoping, Stockholm, Uppsala, Umea, Gothenburg, and Lund see figure 1.

2.2. Patients with chronic pain

The project will mainly use patient-reported data from SQRP (www.ucr.uu.se/nrs/) for the participating university clinics. The project leader (Björn Gerdle) is the research leader for the national research group for SQRP and a member of its steering group.

SQRP is based on *Patient Reported Outcome Measures* (PROM) in the form of a written questionnaire. Validated instruments are used in the survey and collectively capture the various facets of the International Classification of Functional Conditions, Disabilities and Health (ICF). The survey includes background variables (*age, sex, country of origin, education and healthcare consumption, weight, height, pain duration and pain extent (according to a list of 36 anatomical regions)*) and pain characteristics, degree of FM-ness, psychological distress, difficulty sleeping, pain coping, fear of movement, consequences of living with chronic pain, physical activity level, as well as health-related quality of life and perceived health (Table 1, Appendix 1). The response rate is > 90% in SQRP, which can be interpreted as patients considering it important to be able to share how they experience their situation. The current diagnoses according to ICD-10 are also registered in SQRP.

The patients who refer to the clinics respond to the SQRP questionnaire according to table 1 in connection with clinical assessment. Patients participating in IPRP also answer questionnaires immediately after and 12 months after rehabilitation.

All patients who respond to SQRP at the relevant university clinics will be asked to participate in the study and may submit saliva samples and blood samples (see below). In this project we will study the following 11 conditions/diagnoses (based on the manual produced within SQRP by, among others, the project leader):

For peer review only - http://bmjopen.bmj.com/site/about/guidelines.xhtml

1. Chronic neck-shoulder pain (cervicalgia, trapezius myalgia)
2. Chronic low back pain without sciatica
3. Generalized pain (CWP) according to criteria ACR 2016 criteria.
4. Fibromyalgia according to the ACR 2016 criteria.
5. Neuropathic pain including sciatica
6. Chronic Whiplash Disorders (WAD)
7. Ehlers-Danlos Syndrome (EDS) or Hypermobility Syndrome (HMS)
8. Complex Regional Pain Syndrome (CRPS)
9. Pain post-COVID infection
10. Chronic pain, not elsewhere classified (ICD R52.1, R52.2 and R52.9)

In addition, we will investigate the patterns when several pain diagnoses are present simultaneously.

2.3. Healthy controls

Age- and sex-matched healthy control persons will be recruited via advertisements and answer a customized version of the SQRP questionnaire. Saliva and blood samples will be collected. Half of the healthy controls will be asked to submit new samples after about 10 weeks.

2.4. Exclusion criteria for patients and controls

Coagulation disorders with predisposition to bleeding, medication with anticoagulants (low-dose aspirin is permitted), hypersensitivity to anesthetic, serious psychiatric disease (investigator's judgement, e.g., psychosis or suicidal ideation), and difficulties in understanding the Swedish language will be excluded.

2.5. Supplementary survey

In addition to the instruments included in the SQRP (or equivalent adapted for healthy controls), the participants will also answer a brief questionnaire in close connection with the tissue sampling. This charts the time of sampling, fasting or not, current medication and health food preparations (e.g., curcumin), pain intensity, and present and previous illnesses e.g., diabetes.

For peer review only - http://bmjopen.bmj.com/site/about/guidelines.xhtml

For peer review only

2.6. Biofluids sampling

Sampling consists of saliva and blood samples drawn in the morning and during fasting. Saliva samples will be taken with Salivette® (Sarstedt AG & Co, PO Box 1220, D-51582, Nümbrecht, Germany, obtained from VWR, article number: 101093–968). The participants ask to avoid brushing teeth or drinking (water is ok) at least one hour before sampling. They rinse their mouth with water and wait for 15 minutes before taking the swab in the mouth. After 3 minutes, they spit the swab into the salivette tube containing a protease cocktail inhibitor. The salivette will be centrifuged (2 minutes, 1,000 x g) and the supernatant is transferred to a new tube and aliquots into 200 µl in 0.6 ml eppendorf tubes and will be stored at -86 ° C until analysis. The swab will be stored at -86°C for future cell extraction. The total volume of saliva will be recorded together with time of sampling, any complication e.g., bleeding, shorter time than 3 minutes due to un-comfortable feelings to have the swab in the mouth.

Blood samples (2 x 8.5 ml) will be collected in P100 tubes (article number: 366448, BD Diagnostics System, Frankling Lakes, NJ, USA) according to the manufacture's recommendations. The sample will be centrifuged at 2500 g for 20 minutes at room temperature within 2-4 hours. Plasma will be extracted by carefully removing the upper part of the supernatant in fractions to a 10 ml tube, and after mixing gently will be aliquoted into 200 µl in 0.6 ml eppendorf tubes and store at -86°C. The cell fraction will be removed to a new tube and store at -86°C. The time for blood sampling and centrifugation, any signs of hemolysis, any complication with sampling will be recorded.

The samples will be handled according to previously developed methodology [16, 17].

2.7. Biochemical analyses

The analyzes that will be used in this study are mainly exploratory omics analysis, i.e., which proteins, metabolites and lipoproteins will be identified cannot be determined in advance. The following analyzes will be performed according to the methodology previously described by our research group [15, 17, 26].

- Exploratory analyzes using panels for inflammation, cytokines & chemokines and neuroinflammation comprising many proteins (from Olink Bioscience, Uppsala [15] and from Meso Scale Discovery [26].

For peer review only - http://bmjopen.bmj.com/site/about/guidelines.xhtml

- Exploratory proteomics analysis using 2D gel electrophoresis and/or shotgun proteomics [16, 33]
- Exploratory Metabolomic and Lipoprotein profile including free fatty acids (FFAs)
- Anti-nociceptive substances, i.e., endocannabinoids, NAE, endorphins, alpha-amylase, and cortisol.
- Metabolites such as lactate, pyruvate, glutamate, and other single pain-mediating molecules such as substance P, bradykinin, serotonin and BDNF.
- Pending analysis, the samples are stored in the biobank facilities with which the participating university clinics are associated.

2.8. Data from SQRP

The surveys in SQRP, including the supplementary questionnaire, will include a careful characterization of the patients and the healthy controls. In the correlation analyzes between biomarkers and pain characteristics, the following variables will initially be used:

- Diagnosis (from manual produced within SQRP)
- Pain intensity
- Extent of pain spreading/Pain localization
- Pain duration
- Psychologic distress (depression, anxiety, pain catastrophizing, fear of movement and insomnia)
- Sex
- Body mass index (BMI)
- Physical activity level
- Pharmacological therapy and health food preparations

2.9. Interdisciplinary multi modal rehabilitation program (IPRP)

The inclusion criteria will be decided by the clinical examination and screening questionnaires concerning both anthropometric measures as well as subjective health followed by an inter professional conference between the MD, a psychologist, a physiotherapist, and an occupational therapist. The IPRP team make the decision which patients they believe would benefit from the IPRP, in accordance with recommendations

For peer review only - http://bmjopen.bmj.com/site/about/guidelines.xhtml

from the Swedish Agency for Health Technology Assessments and Assessment of Social Services. The patient should be motivated and have the potential for an active change to be included in IPRP. All patients will participate in a specific individual exercise with a physiotherapist (with both relaxation therapy and strengthening specific regions of the body to unburden), individual- and group therapy led by a clinical psychologist (cognitive behavioral therapy, education, coping and mindfulness) as well as environmental work changes from an occupational therapist, working with education and return to work strategies in group of six to nine persons, commonly 20 h per week of group-based activities for 6 to 8 weeks [34, 35]. Pain education (including lectures in basic pain science and pain management) will be offered both for patients as well as for their relatives, friends, and colleagues. For descriptions and content of the Swedish IPRPs see [36-38]

2.10. Statistics

In addition to descriptive statistics, advanced multivariate statistics (MVDA [18]; i.e., advanced principal component analysis (PCA) and OPLS regressions) will be used according to the guidelines presented by Wheelock & Wheelock for omics data [39] using SIMCA®-P + (Sartorius AG, Göttingen Germany). The methods are necessary to manage and take advantage of the intercorrelation pattern (i.e., multicollinearity) between the identified substances. MVDA enables analyzes where the number of variables is significantly greater than the number of observations (i.e., short, and broad data tables), which more traditional multivariate regressions cannot easily handle. The research group has a long experience of the methods and has used these in previous studies and has an established collaboration with statisticians.

2.11 Sample size calculation

There are limited numbers of study that report methods for determining the sample size at MVDA [40]. In the literature, for example, proteomics and metabolomics studies for chronic pain have so far been rather small and usually include 20 + 20 participants. The literature points out the need for large cohorts, especially in complex disease states such as chronic pain, and that the results are replicated in new cohorts. Here we make the assessment that at least 200 patients (from both sexes) in each of the 10 diagnostic groups (see above) must be recruited and the same number for replication of the results. In the group with several diagnoses, the heterogeneity is significant, so double the

For peer review only - http://bmjopen.bmj.com/site/about/guidelines.xhtml

number is deemed necessary. The numbers are required to get a sufficient spread in terms of, for example, psychological load, BMI, and pharmacotherapy and to be able to identify clinically relevant subgroups within a diagnosis. Four hundred healthy controls (even sex distribution) are deemed necessary; half of these leave new samples after about 10 weeks. For analyzes of the relationships with treatment results (mixed diagnoses), 300 patients are considered necessary.

In summary, we intend to recruit a total of 400 patients in each diagnostic group (1-10) and 800 in mixed diagnostic group for analysis at one time, 300 patients participating in IPRP treatment will leave samples immediately before the treatment period and are followed up on two occasions, and 400 healthy controls. This means that 4,800 patients and 400 healthy controls, i.e., a total of 5,200 participants, will be involved.

2.12 Patient and Public Involvement

Neither patients nor the public are involved in the design, conduct, reporting, or dissemination plans associated with this research.

3. Discussion

Sweden has a population of about 10 million people, and the societal costs of approximately 20% of the adult population having moderate-severe chronic pain have been calculated by the Swedish Agency for Health Technology Assessments and Assessment of Social Services in 2003 as SEK 87 billion per year, corresponding to approximatively 1 billion US dollars per million inhabitants and year. Chronic pain is often associated with extensive suffering and poor health. At the same time, it is necessary to state that the effects of, for example, pharmacological treatment are limited; a maximum of 25–30% of the patients report significant and clinically valuable effects. The effect sizes for IPRP are only small to moderate according both to systematic reviews and to registry studies of real-world patients [37, 41].

The importance of developing mechanism-based diagnostics and treatment for chronic pain has been emphasized [42]. Pain clinicians largely lack precision medicine tools that can provide support for treatment choice. This constitutes a significant lack of knowledge about the biological component of the bio-psycho-social model on which modern pain care is expected to rest. This deficiency reasonably helps to explain the

For peer review only - http://bmjopen.bmj.com/site/about/guidelines.xhtml

relatively small effects of treatment and rehabilitation. Our new cooperative multicenter project from all Swedish Universities combined will play a crucial part in the paradigm shift occurring and focusing on a personalized individual treatment method where we will be using advanced metabolomic as well as proteomic methods. The present project is an example of the paradigm shift that is now taking place in modern clinical medicine in a direction towards precision medicine where *omic* research is a crucial element. The new research is characterized by having a focus on biological processes and involves developing a clinical medicine which is based not only on anamnesis and clinical examination but that also includes the possibility to measure the actual pathophysiological mechanism at work in each patient (hence, precision medicine). This is crucial for the development of the clinical diagnosis and treatment of chronic pain and other conditions and diseases in which the clinically oriented researchers participating in this project are involved and engaged.

The project is on an international research front and has great potential to contribute to an improved understanding of activated nociceptive and pain mechanisms and thereby better diagnosis and, in the long term, treatment of chronic pain. It will contribute to the development of clinically useful saliva and blood samples that can be used in the investigation and choice of treatment for patients with chronic pain. In this way, the project will be able to contribute to the development of investigation and treatment that is indeed based on the individual patient's activated nociception and pain mechanisms ("personalized medicine", "precision medicine"). The results will also be able to form the basis for new pharmacological development, helping to bridge the gap between clinical pain medicine and animal models (c.f. translation and back-translation).

The feasibility of the project is very high. The research group has broad and adequately high competence for the implementation. The participating university clinics have working routines for collecting data for SQRP; (response rate> 90%) and routines for recruiting healthy participants. Extensive experience of the biochemical and statistical methods that will be used in the project is available. We have access to all necessary equipment partly within our research laboratory Painomics laboratory at Linköping University, through the Faculty of Medicine's Core Facility at Linköping University and

For peer review only - http://bmjopen.bmj.com/site/about/guidelines.xhtml

the local node in Linköping that is under construction and connected to the Swedish Nuclear Magnetic Resonance spectroscopy (NMR) center in Gothenburg.

In the studies that form the basis of the project and use the same methods, we have found marked differences in the protein/inflammation pattern in muscles and plasma at CWP/FM [16, 17, 33]. In the proteomics studies of musculature and plasma, we have been able to identify proteins that can with great certainty explain the group affiliation (patient or healthy) (R2: 0.81- 0.84) [16, 17]; similar results are obtained with the inflammation panel [15]. The significant proteins in the proteomics studies reflect activated nociceptive, inflammatory, and various metabolic processes. Similar conclusions are drawn in other existing proteomics studies [17, 43-45]. Our proteomics/inflammation research has received attention and been highlighted as innovative and internationally leading [46, 47]. A recently published systematic review identified that proteomics research on chronic pain is dominated by researchers from Sweden (mainly this research group) [48].

Overall, this clinically anchored project will be of great importance for the optimization of the clinical investigation and assessment as well as for better and more adapted treatment interventions based on the activated nociceptive mechanisms. This project will fill an important gap in our knowledge of the molecular mechanisms of common chronic pain conditions in humans and will produce significant steps towards a mechanism based and precision pain medicine.

Ethics

The study is approved by the Swedish Ethical Review Authority (Dnr 2021-04929) and will be conducted in accordance with the declaration of Helsinki. All participants will give their informed consent before the experiments and they will repeatedly be informed about their right to interrupt the participation at any time point, without explanation of their actions. Before data are registered in the database it will be blinded. All measurements are performed by registered health care staff. The participants will receive written information with the telephone number of the project responsible physician to be contacted in case of complications. The visits, any complications, and a summary of the study will be recorded in the electronic record. When taking samples and measuring pain sensitivity, participants may

For peer review only - http://bmjopen.bmj.com/site/about/guidelines.xhtml

For peer review only

experience short periods of (increased) pain. However, this discomfort should not be more extensive than temporary pain that is experienced in everyday situations.

The samples of blood and saliva will be frozen immediately after collection and stored in biobank at; Östergötland, reg nr 1, Örebro biobank, IVO reg nr 454, Biobank Norr, IVO reg nr 472, Uppsala Biobank, IVO reg nr 827, Biobank Väst, IVO reg nr 890, Region Skånes biobank, IVO reg nr 136 and the local biobank at Danderyd hospital AB in Stockholm.

Results will be presented at the group level without the possibility of identifying any single individual. *Overall*, our assessment is that the benefits of this project are considerably greater than the risks.

Contributorship statement

BG,BGh and EB were responsible for conception of this research project. All authors contributed to the study design. BGh and ME draft the first version of the manuscript. MR, OG, and BG were responsible for the communication plan with the steering group at the clinic. EK, HW, ÅR, YFL and BG were responsible for the scientific hypothesis. ME and YFL were responsible for ethical application. E B, ML, HG, KU and KR were responsible for the protocol for clinical procedures to recruit patients. BGh, NS and BS were responsible for the protocol for sampling, sample handling and biobanking. PA, RK and MR are responsible for financing plan for the project.

All authors have been involved in editing, review and accepted the final version of the manuscript.

Competing interests

The authors declare no conflict of interest.

Funding

This work is supported by grants from the Petrus and Augusta Hedlunds Foundation (grant N/A), the Gun and Bertil Stohnes Foundation (grant N/A), the Loo and Hans Osterman Foundation (grant N/A), the Demens foundation (grant N/A), the Brain foundation (grant FO2018-0315), the Senil demens Särfond foundation Region Örebro (grant N/A), the Gamla Tjänarinnor foundation (grant N/A), the Swedish Research Council (grant 2018-02470) and the Research-ALF region Ostergotland (grant RÖ-834951).

For peer review only - http://bmjopen.bmj.com/site/about/guidelines.xhtml

Data sharing statement

The datasets generated during the study will be available from the corresponding author on reasonable request after the final reports have been published.

References

1. Breivik, H., et al., *Survey of chronic pain in Europe: prevalence, impact on daily life, and treatment.* Eur J Pain, 2006. **10**(4): p. 287-333.
2. Bromley Milton, M., et al., *Is Pain Intensity Really That Important to Assess in Chronic Pain Patients? A Study Based on the Swedish Quality Registry for Pain Rehabilitation (SQRP).* PLoS One, 2013. **8**(6): p. e65483.
3. Vos, T., et al., *Years lived with disability (YLDs) for 1160 sequelae of 289 diseases and injuries 1990-2010: a systematic analysis for the Global Burden of Disease Study 2010.* Lancet, 2012. **380**(9859): p. 2163-96.
4. Fillingim, R.B., *Individual differences in pain: understanding the mosaic that makes pain personal.* Pain, 2017. **158 Suppl 1**(Suppl 1): p. S11-s18.
5. Gatchel, R.J., et al., *The biopsychosocial approach to chronic pain: scientific advances and future directions.* Psychol Bull, 2007. **133**(4): p. 581-624.
6. Engel, G.L., *The need for a new medical model: a challenge for biomedicine.* Science, 1977. **196**(4286): p. 129-36.
7. Cleve, M., et al., *Assessment of intra- and inter-regional interrelations between GABA+, Glx and BOLD during pain perception in the human brain - A combined (1)H fMRS and fMRI study.* Neuroscience, 2017. **365**: p. 125-136.
8. Lamichhane, B., et al., *Functional Disruptions of the Brain in Low Back Pain: A Potential Imaging Biomarker of Functional Disability.* Front Neurol, 2021. **12**: p. 669076.
9. Kosek, E., et al., *Chronic nociplastic pain affecting the musculoskeletal system: clinical criteria and grading system.* Pain, 2021. **162**(11): p. 2629-2634.
10. Rodriguez-Raecke, R., et al., *Structural brain changes in chronic pain reflect probably neither damage nor atrophy.* PLoS One, 2013. **8**(2): p. e54475.
11. Gerdle, B., et al., *Chronic musculoskeletal pain: review of mechanisms and biochemical biomarkers as assessed by the microdialysis technique.* J Pain Res, 2014. **7**: p. 313-26.
12. Üçeyler, N., et al., *Small fibre pathology in patients with fibromyalgia syndrome.* Brain, 2013. **136**(Pt 6): p. 1857-67.
13. Gunn, J., et al., *An Analysis of Biomarkers in Patients with Chronic Pain.* Pain Physician, 2020. **23**(1): p. E41-e49.
14. Islam, B., et al., *The Identification of Blood Biomarkers of Chronic Neuropathic Pain by Comparative Transcriptomics.* Neuromolecular Med, 2022. **24**(3): p. 320-338.
15. Gerdle, B., et al., *Signs of ongoing inflammation in female patients with chronic widespread pain: A multivariate, explorative, cross-sectional study of blood samples.* Medicine (Baltimore), 2017. **96**(9): p. e6130.
16. Olausson, P., et al., *Protein alterations in women with chronic widespread pain--An explorative proteomic study of the trapezius muscle.* Sci Rep, 2015. **5**: p. 11894.
17. Wåhlén, K., et al., *Systemic alterations in plasma proteins from women with chronic widespread pain compared to healthy controls: a proteomic study.* J Pain Res, 2017. **10**: p. 797-809.
18. Olausson, P., et al., *Specific proteins of the trapezius muscle correlate with pain intensity and sensitivity - an explorative multivariate proteomic study of the trapezius muscle in women with chronic widespread pain.* J Pain Res, 2016. **9**: p. 345-56.

For peer review only - http://bmjopen.bmj.com/site/about/guidelines.xhtml

19. Walker, A.K., et al., *Neuroinflammation and comorbidity of pain and depression.* Pharmacol Rev, 2014. **66**(1): p. 80-101.
20. Felger, J.C. and F.E. Lotrich, *Inflammatory cytokines in depression: neurobiological mechanisms and therapeutic implications.* Neuroscience, 2013. **246**: p. 199-229.
21. Ernberg, M., et al., *Effects of 15 weeks of resistance exercise on pro-inflammatory cytokine levels in the vastus lateralis muscle of patients with fibromyalgia.* Arthritis Res Ther, 2016. **18**(1): p. 137.
22. Andrés-Rodríguez, L., et al., *Peripheral immune aberrations in fibromyalgia: A systematic review, meta-analysis and meta-regression.* Brain Behav Immun, 2020. **87**: p. 881-889.
23. O'Mahony, L.F., et al., *Is fibromyalgia associated with a unique cytokine profile? A systematic review and meta-analysis.* Rheumatology (Oxford), 2021. **60**(6): p. 2602-2614.
24. Bäckryd, E., et al., *Hepatocyte growth factor, colony-stimulating factor 1, CD40, and 11 other inflammation-related proteins are associated with pain in diabetic neuropathy: exploration and replication serum data from the Pain in Neuropathy Study.* Pain, 2021.
25. Bäckryd, E., et al., *High levels of cerebrospinal fluid chemokines point to the presence of neuroinflammation in peripheral neuropathic pain: a cross-sectional study of 2 cohorts of patients compared with healthy controls.* Pain, 2017. **158**(12): p. 2487-2495.
26. Jönsson, M., et al., *The inflammatory profile of cerebrospinal fluid, plasma, and saliva from patients with severe neuropathic pain and healthy controls-a pilot study.* BMC Neurosci, 2021. **22**(1): p. 6.
27. Rethorst, C.D., et al., *Pro-inflammatory cytokines as predictors of antidepressant effects of exercise in major depressive disorder.* Mol Psychiatry, 2013. **18**(10): p. 1119-24.
28. Lasselin, J., et al., *Low-grade inflammation may moderate the effect of behavioral treatment for chronic pain in adults.* J Behav Med, 2016. **39**(5): p. 916-24.
29. Bujak, B.K., et al., *The effectiveness of interdisciplinary intensive outpatient programs in a population with diverse chronic pain conditions: a systematic review and meta-analysis.* Pain Manag, 2019. **9**(4): p. 417-429.
30. Salathé, C.R., et al., *Treatment Efficacy, Clinical Utility, and Cost-Effectiveness of Multidisciplinary Biopsychosocial Rehabilitation Treatments for Persistent Low Back Pain: A Systematic Review.* Global Spine J, 2018. **8**(8): p. 872-886.
31. Kamper, S.J., et al., *Multidisciplinary biopsychosocial rehabilitation for chronic low back pain.* Cochrane Database Syst Rev, 2014(9): p. Cd000963.
32. Papadopoulou, D., et al., *A meta-analysis to determine the effect of pharmacological and non-pharmacological treatments on fibromyalgia symptoms comprising OMERACT-10 response criteria.* Clin Rheumatol, 2016. **35**(3): p. 573-86.
33. Wåhlén, K., et al., *Proteomic Investigation in Plasma from Women with Fibromyalgia in Response to a 15-wk Resistance Exercise Intervention.* Med Sci Sports Exerc, 2022. **54**(2): p. 232-246.
34. Gerdle, B., et al., *Changes in inflammatory plasma proteins from patients with chronic pain associated with treatment in an interdisciplinary multimodal rehabilitation program - an explorative multivariate pilot study.* Scand J Pain, 2019. **20**(1): p. 125-138.
35. Scascighini, L., et al., *Multidisciplinary treatment for chronic pain: a systematic review of interventions and outcomes.* Rheumatology (Oxford), 2008. **47**(5): p. 670-8.
36. Björn, G., F. Marcelo Rivano, and R. Åsa, *Interdisciplinary Pain Rehabilitation Programs: Evidence and Clinical Real-World Results*, in *Pain Management - From Pain Mechanisms to Patient Care*, W. Dr. Alice, Editor. 2022, IntechOpen: Rijeka. p. Ch. 2.
37. Ringqvist, Å., et al., *Moderate and Stable Pain Reductions as a Result of Interdisciplinary Pain Rehabilitation-A Cohort Study from the Swedish Quality Registry for Pain Rehabilitation (SQRP).* J Clin Med, 2019. **8**(6).
38. Gerdle, B., et al., *Outcomes of Interdisciplinary Pain Rehabilitation Across Subgroups of the Multidimensional Pain Inventory - A Study From the Swedish Quality Registry for Pain Rehabilitation.* Pain Pract, 2021. **21**(6): p. 662-679.

For peer review only - http://bmjopen.bmj.com/site/about/guidelines.xhtml

39. Wheelock Å, M. and C.E. Wheelock, *Trials and tribulations of 'omics data analysis: assessing quality of SIMCA-based multivariate models using examples from pulmonary medicine.* Mol Biosyst, 2013. **9**(11): p. 2589-96.
40. Saccenti, E. and M.E. Timmerman, *Approaches to Sample Size Determination for Multivariate Data: Applications to PCA and PLS-DA of Omics Data.* Journal of Proteome Research, 2016. **15**(8): p. 2379-2393.
41. Elbers, S., et al., *Longitudinal outcome evaluations of Interdisciplinary Multimodal Pain Treatment programmes for patients with chronic primary musculoskeletal pain: A systematic review and meta-analysis.* Eur J Pain, 2022. **26**(2): p. 310-335.
42. Vardeh, D., R.J. Mannion, and C.J. Woolf, *Toward a Mechanism-Based Approach to Pain Diagnosis.* J Pain, 2016. **17**(9 Suppl): p. T50-69.
43. Wåhlén, K., et al., *Significant correlation between plasma proteome profile and pain intensity, sensitivity, and psychological distress in women with fibromyalgia.* Scientific Reports, 2020. **10**(1): p. 12508.
44. Han, C.-L., et al., *Serum proteome profiles revealed dysregulated proteins and mechanisms associated with fibromyalgia syndrome in women.* Scientific Reports, 2020. **10**(1): p. 12347.
45. Ramírez-Tejero, J.A., et al., *Insight into the biological pathways underlying fibromyalgia by a proteomic approach.* J Proteomics, 2018. **186**: p. 47-55.
46. Sommer, C., *Exploring pain pathophysiology in patients.* Science, 2016. **354**(6312): p. 588-592.
47. Gazerani, P. and H.S.H. Vinterhøj, *'Omics': An emerging field in pain research and management.* Future Neurology, 2016. **11**(4): p. 255-265.
48. Gerdle, B. and B. Ghafouri, *Proteomic studies of common chronic pain conditions - a systematic review and associated network analyses.* Expert Rev Proteomics, 2020. **17**(6): p. 483-505.

For peer review only

For peer review only - http://bmjopen.bmj.com/site/about/guidelines.xhtml

Figure legends

Figure 1. Flow chart of the study design outlining numbers of subjects in each chronic pain group and healthy controls.

Tables

Table 1. Patient reported outcome measures to be used in the project. NRS = Numeric Rating Scale, MPI = Multidimensional Pain Inventory Swedish version, HADS = Hospital Anxiety and Depression Scale, PCS = Pain Catastrophizing Scale, ISI = insomnia Severity Index, CPAQ8= Chronic Pain Acceptance Questionnaire based upon 8 items, TSK = Tampa Scale for Kinesiophobia, RAND-36 = modernized version of Short-Form Health Survey-36.

**Survey**	**Instrument/Scales**	**Properties**
Pain characteristics		
Intensity	NRS (0-10)	0 = no pain, 10 = worst pain experienced
	MPI Pain intensity (0-6)	Based on 2 items, each scored 0-6
Interference	MPI-Pain Interference (0-6)	Based on 11 items, each scored 0-6
Fibromyalgia	2016 criteria	Widespread pain index (WPI): 0-19 sites<br>Symptom severity score (SSS): 0-12<br>FMness score: WPI + SSS.
Psychological distress		
Depression	HADS-Depression (0-21)	Sum of 7 items, each scored 0-3
Anxiety	HADS-Anxiety (0-21)	Sum of 7 items, each scored 0-3
	MPI-Affective distress (0-6)	Based on 3 items each scored 0-6
Pain catastrophizing	PCS (0-52)	Sum of 13 items, each scored 0-4; 3 subscales can be obtained
Difficulty sleeping	ISI (0-28)	Sum of 7 items, each scored 0-4
Pain coping	CPAQ8 (0-48)	Sum of 8 items, each scored 0-3; 2 subscales can be obtained
Fear of movement	TSK (0-68)	17 items, each scored 0-4 according to agreement with statement and summed
Impact of chronic pain		
Control of life	MPI-Life control (0-6)	Based on 4 items, each scored 0-6
Social support	MPI-Social support (0-6)	Based on 2 items, each scored 0-6
Health-related quality of life	RAND-36 (0-100)	36 items in 8 subcategories scored varying between 0 and 100% (with fixed intervals). The mean for each subscale is calculated
	EQ-5D™	Consists of two parts. The first part is an index obtained from five dimensions items scored 0-5. The second part is self-estimation of today's health according to a 100-point thermometer-like scale (EQ5D-VAS) with defined end points (high values indicate

For peer review only - http://bmjopen.bmj.com/site/about/guidelines.xhtml

	better health and low values indicate worse health)
Physical activity	
Sedentary behavior	Minutes per week
Low intensity	
Moderate intensity	
Vigorous intensity	

For peer review only

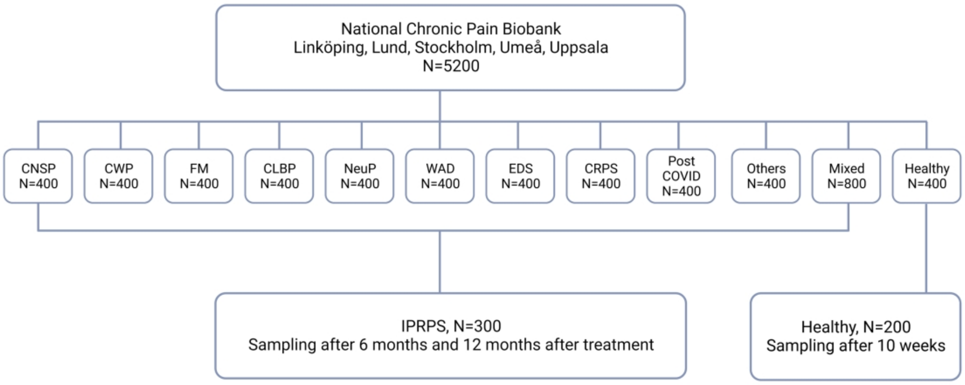

Figure 1. Flow chart of the study design outlining numbers of subjects in each chronic pain group and healthy controls.

258x111mm (300 x 300 DPI)

Appendix 1

Table: References to psychometric characteristics for the patient reported outcome measures (cf. Table 1) to be used in the project.

Instrument/Scale	References
Pain intensity (NRS)	1
Fibromyalgia 2016 criteria	2
Psychological distress (HADS)	3 4
Multidimensional Pain Inventory (MPI)	5-7
Pain catastrophizing (PCS)	8 9
Insomnia (ISI)	10 11
Pain coping (CPAQ8)	12-14
Fear of movement (TSK)	15-18
Health-related quality of life (RAND-36)	19-22
Health-related quality of life (EQ-5D^{TM})	23-26
Physical activity	27 28

NRS = Numeric Rating Scale, MPI = Multidimensional Pain Inventory, HADS = Hospital Anxiety and Depression Scale, PCS = Pain Catastrophizing Scale, ISI = insomnia Severity Index, CPAQ = Chronic Pain Acceptance Questionnaire, TSK = Tampa Scale for Kinesiophobia, RAND-36 = modernized version of Short-Form Health Survey-36.

References

1. Dworkin RH, Turk DC, Farrar JT, et al. Core outcome measures for chronic pain clinical trials: IMMPACT recommendations. *Pain* 2005;113(1-2):9-19. [published Online First: 2004/12/29]
2. Wolfe F, Clauw DJ, Fitzcharles MA, et al. 2016 Revisions to the 2010/2011 fibromyalgia diagnostic criteria. *Semin Arthritis Rheum* 2016;46(3):319-29. doi: 10.1016/j.semarthrit.2016.08.012 [published Online First: 2016/12/06]
3. Lisspers J, Nygren A, Söderman E. Hospital Anxiety and Depression Scale (HAD): some psychometric data for a Swedish sample. *Acta Psychiatrica Scandinavica,* 1997;96(4):281-6.
4. Zigmond AS, Snaith RP. The hospital anxiety and depression scale. *Acta Psychiatr Scand* 1983;67(6):361-70. [published Online First: 1983/06/01]
5. Bergstrom G, Jensen IB, Bodin L, et al. Reliability and factor structure of the Multidimensional Pain Inventory--Swedish Language Version (MPI-S). *Pain* 1998;75(1):101-10. [published Online First: 1998/04/16]
6. Kerns RD, Turk DC, Rudy TE. The West Haven-Yale Multidimensional Pain Inventory (WHYMPI). *Pain* 1985;23(4):345-56. [published Online First: 1985/12/01]
7. Turk D, Rudy T. Toward an empirically derived taxonomy pf chronic pain patients: integration of psychological assessment data. *J Consult Clin Psychol* 1988;56:233-8.
8. Quartana PJ, Campbell CM, Edwards RR. Pain catastrophizing: a critical review. *Expert Rev Neurother* 2009;9(5):745-58. doi: 10.1586/ern.09.34
9. Sullivan M, Bishop S, Pivik J. The Pain catastrophizing scale: development and validation. *Psychol Assess* 1995;7:524-32.

For peer review only

10. Dragioti E, Wiklund T, Alfoldi P, et al. The Swedish version of the Insomnia Severity Index: Factor structure analysis and psychometric properties in chronic pain patients. *Scand J Pain* 2015;9(1):22-27. doi: 10.1016/j.sjpain.2015.06.001 [published Online First: 2018/06/19]
11. Jansson M, Linton SJ. Cognitive-behavioral group therapy as an early intervention for insomnia: a randomized controlled trial. *J Occup Rehabil* 2005;15(2):177-90. doi: 10.1007/s10926-005-1217-9 [published Online First: 2005/04/23]
12. Rovner GS, Arestedt K, Gerdle B, et al. Psychometric properties of the 8-item Chronic Pain Acceptance Questionnaire (CPAQ-8) in a Swedish chronic pain cohort. *J Rehabil Med* 2014;46(1):73-80. doi: 10.2340/16501977-1227
13. Fish RA, McGuire B, Hogan M, et al. Validation of the chronic pain acceptance questionnaire (CPAQ) in an Internet sample and development and preliminary validation of the CPAQ-8. *Pain* 2010;149(3):435-43. doi: 10.1016/j.pain.2009.12.016 [published Online First: 2010/03/02]
14. Vowles KE, McCracken LM, McLeod C, et al. The Chronic Pain Acceptance Questionnaire: confirmatory factor analysis and identification of patient subgroups. *Pain* 2008;140(2):284-91. doi: 10.1016/j.pain.2008.08.012 [published Online First: 2008/10/01]
15. Lundberg M, J. S, SG. C. A psychometric evaluation of the Tampa Scale for Kinesiophobia - from a physiotherapeutic perspective. *Physiotherapy Theory and Practice* 2004;20(2):121 - 33
16. Cleland JA, Fritz JM, Childs JD. Psychometric properties of the Fear-Avoidance Beliefs Questionnaire and Tampa Scale of Kinesiophobia in patients with neck pain. *Am J Phys Med Rehabil* 2008;87(2):109-17. doi: 10.1097/PHM.0b013e31815b61f1
17. Miller R, Kori S, Todd D. The Tampa Scale: a measure of kinesiophobia. *Clin J Pain* 1991;7(1):51-52.
18. Roelofs J, Goubert L, Peters ML, et al. The Tampa Scale for Kinesiophobia: further examination of psychometric properties in patients with chronic low back pain and fibromyalgia. *Eur J Pain* 2004;8(5):495-502. doi: 10.1016/j.ejpain.2003.11.016
19. Sullivan M, Karlsson, J., Ware, J. The Swedish 36 Health survey. Evaluation of data quality, scaling assumption, reliability and construct validity across general populations in Sweden. *Soc Sci Med* 1995;41:1349-58.
20. Hays RD, Morales LS. The RAND-36 measure of health-related quality of life. *Ann Med* 2001;33(5):350-7. doi: 10.3109/07853890109002089 [published Online First: 2001/08/09]
21. Hays RD, Sherbourne CD, Mazel RM. The RAND 36-Item Health Survey 1.0. *Health Econ* 1993;2(3):217-27. doi: 10.1002/hec.4730020305 [published Online First: 1993/10/01]
22. Orwelius L, Nilsson M, Nilsson E, et al. The Swedish RAND-36 Health Survey - reliability and responsiveness assessed in patient populations using Svensson's method for paired ordinal data. *J Patient Rep Outcomes* 2017;2(1):4. doi: 10.1186/s41687-018-0030-0 [published Online First: 2017/01/01]
23. Karlsson JA, Nilsson JA, Neovius M, et al. National EQ-5D tariffs and quality-adjusted life-year estimation: comparison of UK, US and Danish utilities in south Swedish rheumatoid arthritis patients. *Ann Rheum Dis* 2011;70(12):2163-6. doi: 10.1136/ard.2011.153437 [published Online First: 2011/08/24]
24. Cheung K, Oemar M, Oppe M, et al. User Guide. Basic information on how to use EQ-5D 2009 [2.0:[Available from: www.eurogol.org accessed 20120101.
25. Brooks R. EuroQol: the current state of play. *Health Policy* 1996;37(1):53-72.
26. EuroQol. EuroQol: a new facility for the measurement of health-related quality of life. *Health Policy* 1990;16:199-208.
27. Kallings L. Validering av Socialstyrelsens screeningfrågor om fysisk aktivitet Stockholm: The Swedish School of Sport and Health Sciences; 2015 [Available from: www.socialstyrelsen.se accessed 20151202.
28. Olsson S. Studies of physical activity in the Swedish population. The Swedish School of Sport and Health Sciences, 2016.

For peer review only

For peer review only
