# Supplementary material for: Swedish Chronic Pain Biobank: protocol for a multicentre registry and biomarker project
Source: BMJ Open. 2022 Nov 30;12(11):e066834. doi: 10.1136/bmjopen-2022-066834 (PMC9717004; doi:10.1136/bmjopen-2022-066834)
Supplement: Reviewer comments [file bmjopen-2022-066834.reviewer_comments.pdf]

PEER REVIEW HISTORY

BMJ Open publishes all reviews undertaken for accepted manuscripts. Reviewers are asked to complete a checklist review form (**http://bmjopen.bmj.com/site/about/resources/checklist.pdf**) and are provided with free text boxes to elaborate on their assessment. These free text comments are reproduced below.

ARTICLE DETAILS

**TITLE (PROVISIONAL)**	The Swedish Chronic Pain Biobank - protocol for a multicenter registry and biomarker project
**AUTHORS**	Ghafouri, B; Ernberg, Malin; Andre'll PhD, Paulin; Bäckryd, Emmanuel; Fisher, Marcelo Rivano; Freund-Levi, Yvonne; Grelz, Henrik; Gräbel, Olaf; Karlsten, Rolf; Kosek, Eva; Löfgren, Monika; Ringqvist, Åsa; Rudling, Karin; Stålnacke, Britt-Marie; Sörlén, Niklas; Uhlin, Karin; Westergren, Hans; Gerdle, Björn

VERSION 1 – REVIEW

**REVIEWER**	Babatunde, Folarin<br>McMaster University, Rehabilitation Science
**REVIEW RETURNED**	16-Sep-2022

**GENERAL COMMENTS**	The study protocol for "The Swedish Chronic Pain Biobank" study was very insightful and offers a noble effort to understand and better manager chronic pain. The team appears well equipped to take on this task and this study would make a great contribution to the literature.<br><br>See comments below to improve the manuscript quality.<br><br>General comments<br>- Review manuscript and ensure the write is completed in future tense since this a protocol.<br>- Review sentence structure to ensure sentences are not too lengthy and unclear.<br>- Include a figure highlighting the flow of participants through the study with focus on assessments, timelines etc.<br>- Include an Appendix highlighting the psychometric properties of the outcome measures propose for use in this study.<br>- Include an Appendix highlighting a basic summary of the interventions proposed in this study. The ref [34] is from 2008 and not adequate to support the validity of the interventions.<br><br>Abstract<br>Line 7: change has to "have"<br>Line 57: paradigm shift in...?<br><br>Introduction<br>Line 11: second sentence is too long.<br>Line 25: Rephrase: "chronic pain and in parallel depression"<br>Line 28: "Depression in turn intensifies the pain by pain" unclear.<br>Lines 21 to 30: Provide supporting references for the first 2 sentences in the second paragraph.<br>Line 49: Rephrase to change sentence structure. A bit convoluted as written.

	Line 3 page 5: "Physical exercise provides anti-inflammatory effects." Purpose of adding this sentence is unclear.<br><br>Methods<br>Describe the method chosen for this study.<br>Clarify the use of the term "sex" as described in the methods versus "gender" as described in the introduction. What construct is this study exploring?<br>Line 54 (page 9): Provide more information on the "brief questionnaire" described. An Appendix would suffice.<br>Line 9 (page 10): Provide a reference for the pain education that will be provided.<br>Line 42 (page 12): Are there studies that address or raise some of the questions related to sample size calculation for MVDA.

**REVIEWER**	McHugh, Patrick<br>University of Huddersfield, Pharmacy
**REVIEW RETURNED**	07-Oct-2022

**GENERAL COMMENTS**	The current article entitled 'The Swedish Chronic Pain Biobank - protocol for a multicenter registry and biomarker project' is well presented and highlights an important project in the context of the urgent need for chronic pain biomarkers. The authors have highlighted the importance of reducing the risk of sample heterogeneity, which is extremely difficult in chronic pain studies. Below are some minor comments/questions.<br><br>Minor comments/questions<br><br>1) In the exclusion criteria - should the exclusion include overarching diseases that would have an impact on blood molecular profiles, such as cancer, diabetes, MS etc.<br>2) Why collecting plasma and not other samples e.g. RNA, PBMCs etc.? This could maximise the potential for biomarker discovery, and whilst the patients are present, maximise the study. This could be beneficial downstream in interpreting the functionality of perturbed changes from the plasma profiles.<br>3) The authors highlight that current medication information will be recorded, would medication history not be relevant too, trial and error etc.?, will dosages etc. be collected?<br>4) A quick search of 'chronic pain blood biomarker studies' highlights several papers that have not been included, these should be included.

VERSION 1 – AUTHOR RESPONSE

Comments from reviewer #1	Our reply
The study protocol for "The Swedish Chronic Pain Biobank" study was very insightful and offers a noble effort to understand and better manager chronic pain. The team appears well equipped to take on this task and this study would make a great contribution to the literature.	Thank you for your positive feedback and valuable comments!
General comments	Point taken! The entire manuscript has been revised now.

- Review manuscript and ensure the write is completed in future tense since this a protocol.<br>- Review sentence structure to ensure sentences are not too lengthy and unclear.<br><br>- Include a figure highlighting the flow of participants through the study with focus on assessments, timelines etc.<br><br>- Include an Appendix highlighting the psychometric properties of the outcome measures propose for use in this study.<br><br>- Include an Appendix highlighting a basic summary of the interventions proposed in this study. The ref [34] is from 2008 and not adequate to support the validity of the interventions.	Point taken! An extensive revision has been made.<br>Point taken! We have included a flow chart in the revised manuscript.<br><br>Point taken. This now reported in Appendix 1. Please also note that we have revised Table 1 since unfortunately a preliminary version was included in the original submission. Minor revisions is also made in section 2.2 due to the updated Table 1.<br><br>Point partly taken. We agree that reference 34 (Scascighini et al) is somewhat old but in our opinion still relevant. We have added references in detail describing the content of Swedish interdisciplinary pain rehabilitation programmes.
Abstract<br>Line 7: change has to "have"<br>Line 57: paradigm shift in...?<br><br>Introduction<br>Line 11: second sentence is too long.<br><br>Line 25: Rephrase: "chronic pain and in parallel depression"<br><br>Line 28: "Depression in turn intensifies the pain by pain" unclear.<br><br>Lines 21 to 30: Provide supporting references for the first 2 sentences in the second paragraph.<br><br>Line 49: Rephrase to change sentence structure. A bit convoluted as written.<br><br>Line 3 page 5: "Physical exercise provides anti-inflammatory effects." Purpose of adding this sentence is unclear.	Point taken. Lines 7 has been revised. Line 57 is not valid anymore as this part has been revised according to the Editorial comments, see above.<br>Point taken. this sentence has been revised.<br><br>Point taken! this has been changed.<br><br>Thank you! This sentence has been revised now. *Moreover, positive correlations exist between depressive symptoms and pain intensity*<br><br>Point taken. A reference (13) has been included in the revised manuscript.<br><br>Point taken. the sentence has been revised.<br><br>Point taken. We agree with the reviewer and have removed this sentence in the revised manuscript.
Methods<br>Describe the method chosen for this study.	Point taken.<br>The manuscript has been revised and the word sex is used throght the entire text.

Clarify the use of the term "sex" as described in the methods versus "gender" as described in the introduction. What construct is this study exploring?<br><br>Line 54 (page 9): Provide more information on the "brief questionnaire" described. An Appendix would suffice.<br><br>Line 9 (page 10): Provide a reference for the pain education that will be provided.<br><br>Line 42 (page 12): Are there studies that address or raise some of the questions related to sample size calculation for MVDA.	Point taken. Table 1 has been revised now and more information about questionnarie is added. We have clarified line 54, page 9.<br><br>Point taken! References has been added as it has mentioned in our reply to your generell comments. Unfortunatlly we can not find the sentence about pain education in Line 9 page 10.<br><br>Point taken! We have included reference (35) in the revised manuscript.
**Comments from reviewer #2**	**Our reply**
The current article entitled 'The Swedish Chronic Pain Biobank - protocol for a multicenter registry and biomarker project' is well presented and highlights an important project in the context of the urgent need for chronic pain biomarkers. The authors have highlighted the importance of reducing the risk of sample heterogeneity, which is extremely difficult in chronic pain studies. Below are some minor comments/questions.	Thank you for your positive feedback and valuable comments!
1) In the exclusion criteria - should the exclusion include overarching diseases that would have an impact on blood molecular profiles, such as cancer, diabetes, MS etc.	Thank you for this thoughtful comment. This biobank project will recruit patients from the *Swedish Quality Registry for Pain Rehabilitation* (SQRP). Please note that SQRP is a chronic pain registry, (mostly musculoskeletal) not a cancer pain registry. Hence, including cancer patients, or indeed patients with a progressive severe disease such as MS, is not an issue in this project. We have carefully thought through the exclusion criteria and think it is important not to expand them unnecessarily.<br><br>Concerning diabetes, its presence or absence is recorded in the supplementary survey as per section 2.5.
2) Why collecting plasma and not other samples e.g. RNA, PBMCs etc.? This could maximise the potential for biomarker	We appologize for this. We collect blood samples and store both fractions after centrifugation. This

discovery, and whilst the patients are present, maximise the study. This could be beneficial downstream in interpreting the functionality of perturbed changes from the plasma profiles.	information has been clarified in the revised manuscript.
3) The authors highlight that current medication information will be recorded, would medication history not be relevant too, trial and error etc.?, will dosages etc. be collected?	Thank you for this proposal. Medication history is of course important in clinical practice. However, on balance, we do not think that this data is important enough to warrant registration in the biobank. We have thought through this issue previously and decided to record **concomitant** medication and not medication history. The reviewer's comment gave us the opportunity to think this through again, and we would like to stick to our original plan.<br>The most important reason for collecting medication data is to be able to investigate if there is a confounding effect of concomitant medication on biomarker findings. This registration is in itself quite complicated and work-intensive as we will register not only concomitant drugs but also dosage. Medication history would add a further dimension of complexity, including the questions of how far back in time one should go, and of what really counts as a medication trial or not – hence, the validity and reliability of medication history data would be questionable.<br>On balance, we therefore choose to focus on concomitant medication and hope the reviewer will accept our rationale for this.
4) A quick search of 'chronic pain blood biomarker studies' highlights several papers that have not been included, these should be included.	Point taken! We have revised the referenslist and two references (21 and 23) have been added.

VERSION 2 – REVIEW

**REVIEWER**	McHugh, Patrick<br>University of Huddersfield, Pharmacy
**REVIEW RETURNED**	16-Nov-2022

**GENERAL COMMENTS**	I am happy with the author's responses
